# Highlights in Cysteine Protease Inhibitors as Antiparasitic Agents Against Trypanosomatids

**DOI:** 10.3390/ijms27198861

**Published:** 2026-10-04

**Authors:** Beatriz M. R. Querido, Maria do Céu Sousa, Jorge A. R. Salvador

**Affiliations:** 1 Laboratory of Pharmaceutical Chemistry, Faculty of Pharmacy, University of Coimbra, Pólo das Ciências da Saúde, Azinhaga de Santa Comba, 3000-548 Coimbra, Portugal; uc2021240476@student.uc.pt; 2 Laboratory of Microbiology, Faculty of Pharmacy, University of Coimbra, Pólo das Ciências da Saúde, Azinhaga de Santa Comba, 3000-548 Coimbra, Portugal; 3 CNC-UC—Center for Neuroscience and Cell Biology, University of Coimbra, Rua Larga, Edificio Faculty of Medicine, University of Coimbra, 3004-504 Coimbra, Portugal; 4 CIBB—Center for Innovative Biomedicine and Biotechnology, University of Coimbra, 3004-504 Coimbra, Portugal

**Keywords:** cysteine protease inhibitors, *Trypanosoma cruzi*, *Trypanosoma brucei*, *Leishmania* spp., cruzain, TbrCATL, CPB

## Abstract

*Trypanosoma cruzi*, *Trypanosoma brucei* and *Leishmania* spp. are the causative agents of Chagas disease, human African trypanosomiasis, and leishmaniasis, respectively. These three neglected tropical diseases affect millions of individuals globally, and current therapeutic options remain severely limited. The major cysteine proteases (CPs) of these parasites play critical roles in their infectivity and survival, making them subjects of prolonged research. Therefore, cruzain, TbrCATL and CPB from *Leishmania* spp. have been recognized as the primary targets of most drugs under investigation. Among the diverse chemical classes investigated as cathepsin L-like inhibitors, natural compounds derivatives (including compounds derived from curcumin, Gallinamide A, and flavonoids) as well as molecules containing Michael acceptors, and nitrile and thiosemicarbazone moieties, have emerged as particularly promising scaffolds. The aim of this review is to provide an overview of the advances made over the last decade in the discovery and optimization of CP inhibitors with antitrypanosomal and antileishmanial activity, emphasizing their chemical scaffolds, biological activities, and therapeutic potential.

## 1. Introduction

*Trypanosoma cruzi*, *Trypanosoma brucei*, and *Leishmania* spp. are the causative agents of severe diseases classified as neglected tropical diseases (NTDs) [1]. *T. cruzi* and *T. brucei* primarily affect populations living in rural and impoverished areas of Latin America and Africa, respectively [2,3]. However, the geographical distribution of *T. cruzi* has been expanding into non-endemic regions, principally due to migration patterns [4,5]. As a result, the United States has now documented local transmission of Chagas disease and its endemic status is under evaluation [6]. Regarding *T. brucei*, only a limited number of cases have recently been documented outside of Africa [7,8]. In contrast, *Leishmania* spp. were never limited to a single continent, with regions across America, Africa, Asia and Europe being considered endemic. Nevertheless, the disease is still progressing to non-endemic zones [9,10,11,12].

**Figure 1:**
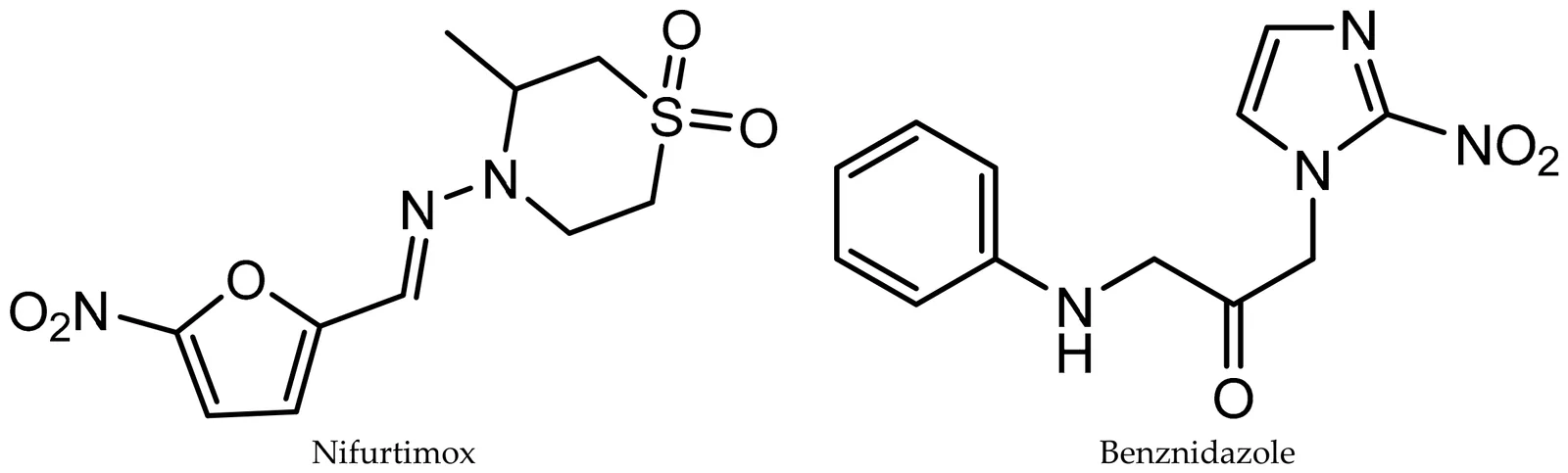
Chemical structure of nifurtimox and benznidazole.

*T. cruzi* is responsible for Chagas disease, an illness that puts at risk more than 100 million people and for which there is no available vaccine [2,13,14,15]. The current treatment consists of benznidazole and nifurtimox (Figure 1) [2,16]. However, both compounds present substantial limitations regarding their efficacy, safety and increasing resistance [17,18,19,20].

*T. brucei* is the causative agent of sleeping sickness, a disease that caused destructive epidemics in Africa and that affects not only people but also livestock animals, contributing to the poverty lived in this continent [21,22]. The prescribed therapy depends on both the stage of the disease and the *Trypanosoma* species responsible for the infection. For *T. brucei gambiense*, it comprises pentamidine, eflornithine, nifurtimox and fexinidazole, all of which have adverse effects such as renal, cardiac and metabolic alterations, besides myelosuppression [3,23,24]. On the other hand, for *T. brucei rhodesiense* the available drugs recommended by the World Health Organization (WHO) are suramin and melarsoprol [3]. DNA-based vaccines are still under investigation, but at this point none of them have reached the market [25,26].

*Leishmania* is a genus comprising different species responsible for the three main clinical forms of leishmaniasis—visceral (VL), cutaneous (CL) and mucocutaneous (ML)—with visceral leishmaniasis (kala-azar) being fatal in up to 95% of the cases left untreated [9,26]. Leishmaniasis is considered an emerging zoonosis, associated with up to 70,000 annual deaths [27,28]. The available treatment is parenteral and oral chemotherapy, which is associated with significant side effects, economical efforts and variable efficacy rates [29,30,31]. Even though numerous initiatives have been undertaken, there is no human-directed vaccine approved yet, unlike in veterinary medicine [32,33]. Nevertheless, veterinary vaccines remain important, since they can reduce the infection in dogs and the human probability of being infected [33,34].

Considering the limitations of the current treatment, the high number of people at risk, and the severe complications associated with these diseases, it is crucial to find new selective and safe drugs against kinetoplastid parasites, as well as new technologies that can facilitate the drug delivery [35,36,37].

Since the beginning of proteomic studies, the major classes of proteases and their role in parasite survival and infection have been extensively investigated [38]. Among serine, threonine, aspartyl, and metallopeptidases, cysteine proteases (CPs) have been the most extensively studied [39,40].

Cruzain is a CP and the most widely studied drug target against *T. cruzi* since the parasite needs it to survive within the host cells [41,42,43,44]. Rhodesain and brucipain, belonging to *T. brucei rhodesiense* and *T. brucei brucei*, respectively, are also CPs necessary for parasite survival, since their inhibition is sufficient to kill the trypomastigote form of *T. brucei* [45,46]. For *Leishmania* spp., even though cathepsin L-like has been receiving more scientific attention than cathepsin B-like, simultaneous inhibition could be a more effective strategy in parasite eradication [47,48,49,50]. Consequently, inhibitors of these enzymes represent a highly promising therapeutic approach against these NTDs [44,51,52].

Given the importance of CPs for parasite survival and pathogenesis, several comprehensive reviews have addressed this topic, covering currently available therapeutics, the existing knowledge of CPs in *T. cruzi*, and *Leishmania* spp., as well as the main classes of CP inhibitors [53,54,55,56]. However, significant advances have been made in the development of selective inhibitors targeting key CPs over the past decade, highlighting the need for an updated overview of the field. Therefore, this review aims to summarize the major advances in the development of selective inhibitors against the principal CPs of *T. cruzi*, *T. brucei* and *Leishmania* spp. over the last decade, with particular emphasis on compounds developed in recent years that were not covered in previous reviews. In addition to providing an overview of the most important selective inhibitors targeting key CPs in these parasites, our review discusses the parasites’ life cycle and the biological relevance of targeting CPs for the development of new therapies against trypanosomatids infections. Furthermore, the approaches employed to improve the efficacy, selectivity, and safety of the compounds currently under investigation are discussed, providing an integrated perspective on recent progress and ongoing challenges in CP-targeted drug discovery.

## 2. *Trypanosoma cruzi* and Chagas Disease

**Figure 2:**
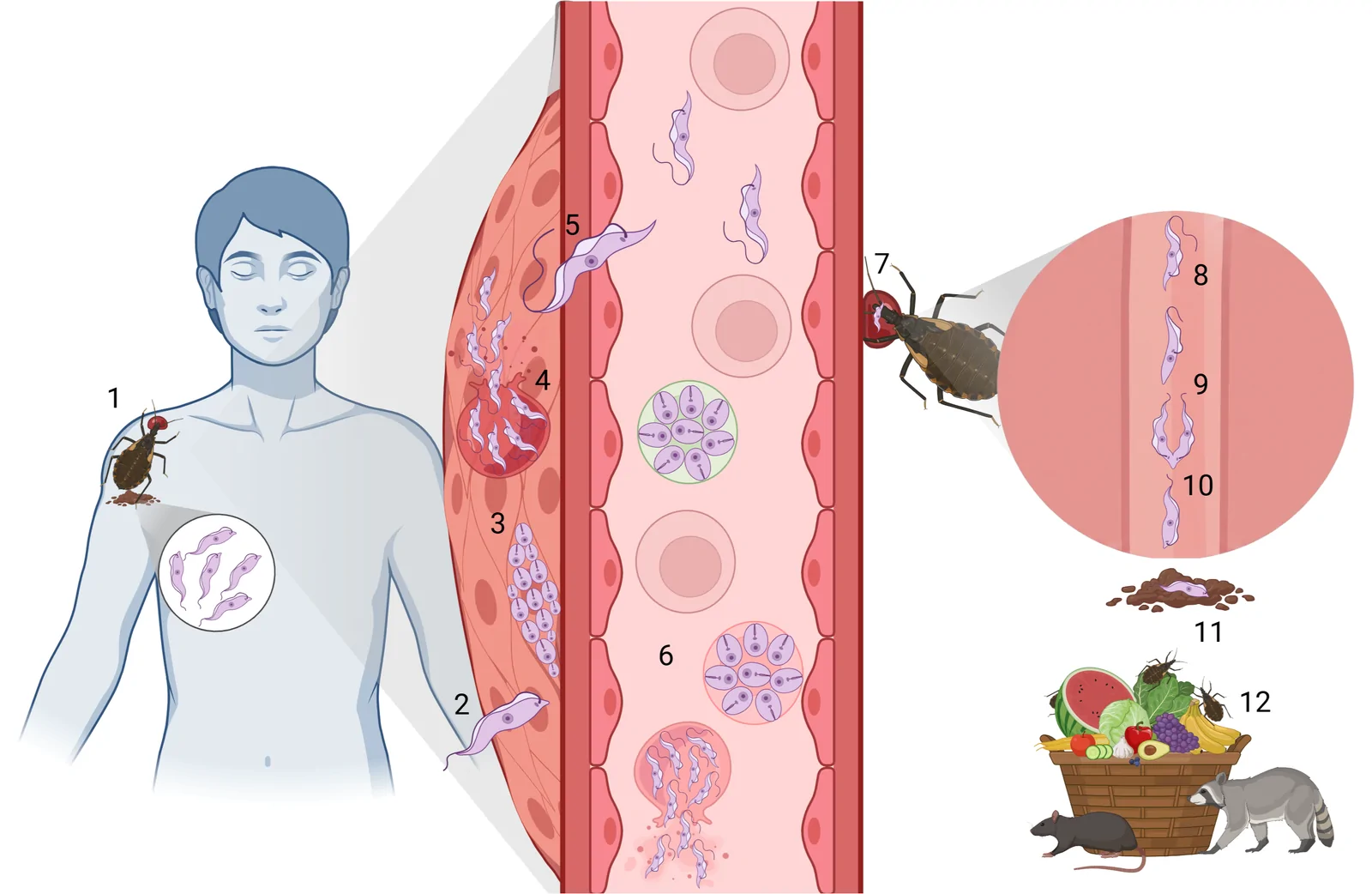
Life cycle of *Trypanosoma cruzi*: (**1**) Infected triatome takes a bloodmeal and metacyclic trypomastigotes present in the insect feces are deposited in the human organism; (**2**) metacyclic trypomastigotes penetrate the organism of the host; (**3**) trypomastigotes differentiate into amastigotes and multiply inside of the human cells; (**4**) the cells burst and amastigotes that have differentiated into trypomastigotes are released and invade the bloodstream (**5**) being able to infect additional cells of the host, disseminating the infection (**6**); (**7**) bloodstream trypomastigotes can be carried in a subsequent blood meal taken by triatomes; (**8**) in the midgut of the insect, trypomastigotes transform into epimastigotes that multiply by binary fission (**9**); (**10**) the epimastigotes migrate toward the hindgut, differentiating into metacyclic trypomastigotes that are subsequently excreted in the feces of the triatomine (**11**); (**12**) metacyclic trypomastigotes can be found in contaminated food, while raccoons and rats are known reservoirs of the parasite, representing additional routes of disease transmission. Created in BioRender. Querido, B. (2026) https://BioRender.com/au68ypr.

In the human host, metacyclic trypomastigotes of *T. cruzi* enter the organism through contamination of a wound or adjacent mucosa (Figure 2). After penetrating cells that surround the bite site, trypomastigotes differentiate into amastigotes that multiply within the cell. Then, the transition from amastigotes to trypomastigotes occurs, and the host cell ruptures, leading to the release of trypomastigotes, capable of invading the bloodstream. Trypomastigotes can be carried in a subsequent blood meal taken by triatomines, differentiating in the midgut of the insect into epimastigote. In the hindgut, epimastigotes are converted into metacyclic trypomastigotes that are excreted in the feces, completing the transmission cycle [39,57]. Mammals can serve as reservoirs of the parasite and transmission can also occur by ingesting food contaminated with triatomine feces, including acai [58,59,60,61,62]. *T. cruzi* can also be transmitted between hosts through blood transfusion, organ transplantation or congenital transmission [63,64,65].

In Chagas disease, the acute phase of the infection is frequently asymptomatic or nonspecific, being characterized by parasitemia in the bloodstream, an inflammatory response and a duration of 4 to 8 weeks [57,66]. If correctly treated, the elimination of the parasite can be successful. If not, it can evolve into a chronic phase, in which parasitemia is totally undetectable. The symptoms of this phase may only appear 10 to 20 years after the infection occurred and can include not only cardiomyopathy, but also alterations in the size and function of the digestive tube, such as megacolon and megaesophagus [57]. It can also affect the neurological system [67].

## 3. *Trypanosoma brucei* and Sleeping Sickness

**Figure 3:**
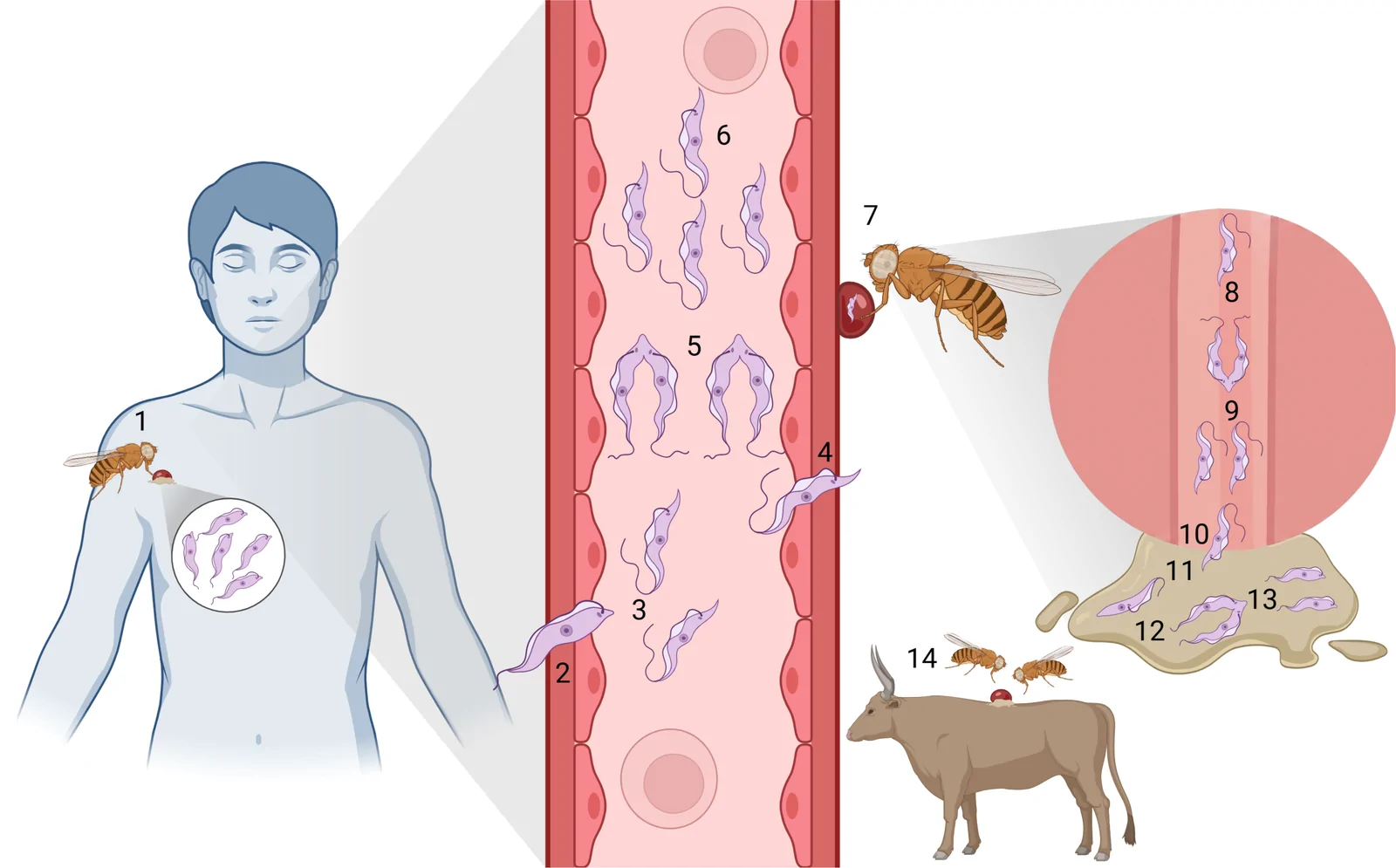
Life cycle of *Trypanosoma brucei:* (**1**) Infected tsetse fly takes a bloodmeal and metacyclic trypomastigotes present in the insect saliva are injected in the human organism; (**2**) metacyclic trypomastigotes penetrate the organism of the host; (**3**) metacyclic trypomastigotes differentiate into bloodstream trypomastigotes and can invade other systems (**4**); (**5**) trypomastigotes multiply in different body fluids, spreading the infection (**6**); (**7**) bloodstream trypomastigotes can be carried in a subsequent blood meal taken by the tsetse fly; (**8**) in the midgut of the insect, trypomastigotes differentiate into procyclic trypomastigotes that multiply by binary fission (**9**); (**10**) then, procyclic trypomastigotes leave and differentiate into epimastigotes (**11**) that multiply in the salivary gland of the insect (**12**) and differentiate into metacyclic trypomastigotes (**13**); (**14**) cattle and wild animals are known reservoirs of the parasite. Created in BioRender. Querido, B. (2026) https://BioRender.com/n1gqw3i.

Of the recognized *T. brucei* species, *T. brucei rhodesiense* and *T. brucei gambiense* are the most important subspecies from a clinical perspective, considering them as the cause of human sleeping sickness [8,22]. Although *T. b. rhodesiense* can infect humans due to its ability to resist the trypanolytic activity of apolipoprotein LI, the resulting disease, in contrast with the one caused by *T. b. gambiense*, is zoonotic rather than anthropogenic, with humans acting as accidental hosts. Consequently, the infection caused by *T. b. rhodesiense* represents only 8% of the reported cases of sleeping sickness [3,68]. Human African trypanosomiasis (HAT) is another denomination for this vector-borne disease mainly transmitted by the bite of *Glossina* species. During the blood feeding, the tsetse fly injects the metacyclic trypomastigote form of *T. brucei* (Figure 3) [39,69]. Following transmission, the metacyclic trypomastigotes differentiate into trypomastigotes that multiply in the bloodstream, the lymphatic circulation and in the spinal fluid, affecting the central nervous system [39]. When the insect takes a blood meal from an infected individual, trypomastigotes are ingested and transformed into procyclic trypomastigotes. Then, the epimastigotes proliferate in the salivary gland and undergo further differentiation into the infective form for the human host [21].

Sleeping sickness progresses through two distinct stages—a hemolymphatic stage, during which the parasite invades the bloodstream and the lymphatic system, causing intermittent fever and lymph node enlargement, and a meningoencephalitic stage, in which trypomastigotes cross the blood–brain barrier (BBB) and invade the brain and cerebrospinal fluid, resulting in characteristic somnolence. Without appropriate treatment, the infection is frequently fatal, and death can be expected after less than a year [8,21].

## 4. *Leishmania* spp. and Leishmaniasis

Leishmaniasis is primarily transmitted through the bite of infected females sand flies of genera *Phlebotomus* and *Lutzomyia*. The parasite life cycle comprises two forms: the intracellular amastigote, present in mammals, and the extracellular form—promastigotes—only found in the human host in the moment of the infection and in the insect [70].

**Figure 4:**
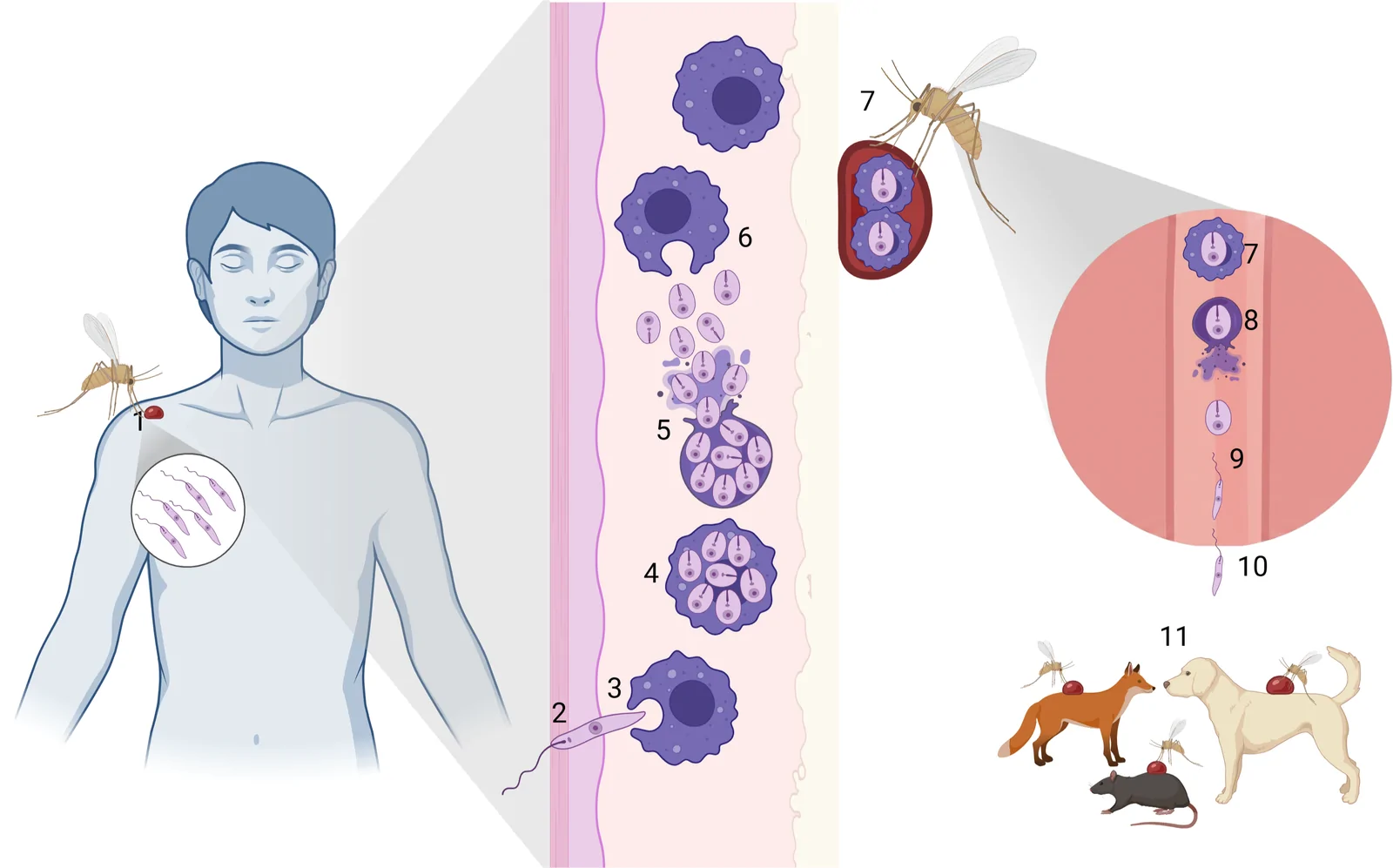
Life cycle of *Leishmania* spp.: (**1**) Infected sand fly takes a bloodmeal and promastigotes are deposited in the human organism, penetrating the skin membrane (**2**); (**3**) the immune system cells phagocyte the promastigote, leading to its differentiation into amastigotes (**4**) that multiply inside macrophages; (**5**) the cells burst and amastigotes are released, being captured by additional cells of the host, disseminating the infection (**6**); (**7**) infected cells can be carried in a subsequent blood meal taken by the sand fly; (**8**) in the gut of the insect, the cells burst and amastigotes differentiate into promastigotes (**9**) that multiply and are carried to the proboscis (**10**); (**11**) domestic and wild animals are known reservoirs of the parasite. Created in BioRender. Querido, B. (2026) https://BioRender.com/3qpzt0e.

During the blood meal, the sandfly first inoculates the promastigote form of *Leishmania* spp. that is rapidly internalized by phagocytic cells, transforming into amastigotes, which multiplies inside the host macrophages (Figure 4) [29,70]. The transmission cycle is completed when the female insect ingests infected host cells during a subsequent blood meal and, in the insect gut, the amastigotes are released and transformed into the promastigote form [70].

VL is associated with a high rate of mortality due to infection of vital internal organs belonging to the reticuloendothelial system, such as the liver, the spleen and even the bone marrow [71,72]. In contrast, CL primarily causes ulcers in the skin and lesions that lead to necrosis, even though it can also affect the lymphatic system [72]. ML affects not only the skin but also mucous membranes and underlying connective tissues [72,73].

## 5. Cysteine Proteases in Trypanosomatids

The discovery of new drugs for NTDs, particularly Chagas disease, HAT and leishmaniasis, remains a major challenge because many candidate compounds exhibit insufficient efficacy or unacceptable toxicological profiles [74,75,76,77]. Moreover, the potential for the emergence of resistance to the current therapies should be considered during the development of new antiparasitic drugs [78].

Of the established molecular targets, CPs, including cruzain in *T. cruzi* and cathepsin L-like and cathepsin B-like proteases in *T. brucei* (TbrCATL and TbrCATB, respectively) and in *Leishmania* spp. (CPA and CPB, and CPC, respectively), have been the focus of several studies due to their role in parasite survival and virulence [56,79]. Furthermore, the architecture of their active sites, together with their structural differences from human cathepsins, highlights the potential of these enzymes as targets for rational drug development [80,81,82].

### 5.1. Cruzain in T. cruzi

#### 5.1.1. Structural and Genomic Features

The *T. cruzi* genome comprises genes that codify four sub-types of cruzipain. Among these, cruzipain-1 and cruzipain-2 are the most extensively characterized [83,84].

**Figure 5:**
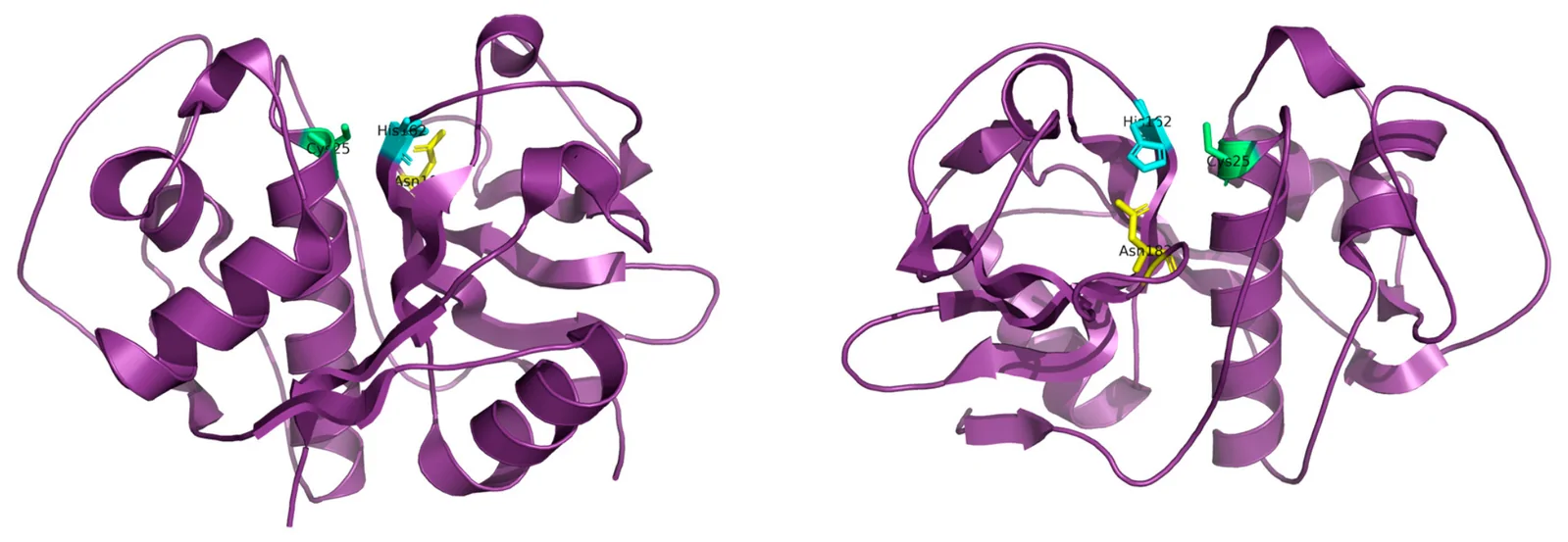
Structural representation of cruzain highlighting its active site, comprising the catalytic His162 (cyan), Cys25 (lime green), and Asn182 (yellow) residues. Structure figures were prepared in PyMOL (version 4.6.0) (PDB ID: 2OZ2) removing the second protein chain (chain C), the bound inhibitors, water molecules and sulfate ions.

Cruzain structure is rigid and stable with its catalytic site (site 1) being composed of three principal aminoamides—His, Cys and Asn (Figure 5) [74,85]. The enzyme belongs to the papain family having two domains and being characterized by its extra carboxyl-terminal chain [83,86]. Due to its proteolytic activity, some potent inhibitors act like its substrate, leading to a rearrangement in the catalytic site of the enzyme [87,88].

Alvarez and colleagues predicted the location of seven allosteric sites in cruzain [85]. Sites 2 and 3 are particularly being pursued for inhibition bonds, with site 3 being reported as the most promising one because its interactions with the selected compounds were able to form stable complexes [85,88]. There is also evidence of non-active sites responsible for the structure of the enzyme, expanding the possibilities of allosteric modulation, an important approach associated with more specificity and selectivity toward the parasite [89,90,91].

Cruzain is found more concentrated in reservosomes between the kinetoplast and the nucleus of the epimastigote form of *T. cruzi* [86]. However, it can also be found in other lysosomes, at the cell surface (contacting directly with the host) and at the flagellar pocket [56,92].

#### 5.1.2. Biological Functions—Physiological Importance

The genes that codify for cruzipain-1 are expressed in all stages of the life cycle of the parasite and *T. cruzi* needs it not only to invade but also to survive and grow inside the host cells [39,41,42,43,44,93,94,95]. Studies demonstrate that cruzain is fundamental for the differentiation of the parasite in the vector and for *T. cruzi* nutrition [86,96]. Additionally, it is associated with infectivity, enabling the increase in vascular permeability and leading to TGF-β pathway activation [95,97,98]. The enzyme further allows parasites to escape the immune system response, since its proteolytic activity targets the hinge region of human IgG and blind the complement, phagocytosis and the immune response drove by antibodies [99,100].

#### 5.1.3. Cruzain as a Therapeutic Target

It is known that when the endogenous cruzain is suppressed by inhibitors, the parasite is unable to complete its life cycle, validating the enzyme as a drug target [39,40,101].

Due to its biological importance, a lot of effort has been put into finding selective cruzain inhibitors. However, the high degree of homology between cruzain and mammalian cathepsin L-like proteases remains the primary challenge [76,81,93]. Finding predictive thermodynamic parameters, like ΔGb, which can be associated with selectivity against the enzyme, can be important to facilitate the development of safer inhibitors [102]. Further characterization of the surface of cruzain and molecular docking studies can also contribute to find selective inhibitors and allosteric modulators or even be an interesting help to design bi-functionalized molecules, such as Proteolysis Targeting Chimeras (PROTACs), a system capable of not only inhibiting the enzyme but of degrading it [103,104,105,106,107]. This strategy, currently being mainly used for cancer drug design, could have an important role in treating diseases caused by trypanosomatids [108,109]. Moreover, cruzain, as the main target of *T. cruzi*, can be the chosen protein of interest (POI) for the development of the first TrypPROTAC [106,110].

### 5.2. Cathepsin L-like and Cathepsin B-like in Trypanosoma brucei

Rhodesain and brucipain in *T. brucei* are cathepsin L-like proteases. They consist of a group of CPs present in *T. brucei*, named according to the subspecies of the parasite [39,110,111]. On the other hand, TbrCATB is a cathepsin B-like from the same parasite, with some structural features similar to cruzain and TbrCATL [56]. However, to the best of our knowledge, no significant progress has been reported in the development of new inhibitors targeting this enzyme over the past decade. Consequently, this review focuses on TbrCATL, which has emerged as the more extensively investigated and therapeutically relevant target.

#### 5.2.1. Structural and Genomic Features of TbrCATL

Rhodesain is a lysosomal protease present in all stages of the *T. brucei rhodesiense* life cycle, which became its principal target. In the same way, brucipain is the principal pursuit target of *T. brucei brucei* [39,110,111,112]. As happens with cruzain, His, Cys and Asn are the three amino acids involved in the active site of TbrCATL [74].

Consistently, TbrCATL and cruzain are first synthesized as proenzymes that are later activated as needed, further supporting their structural and functional similarity [113,114]. However, this zymogen (pro-TbrCATL) has been recently studied and revealed to have a unique alteration in its core protease domain, which can be used to find new selective anti-pro-TbrCATL drugs [114]. The main structural difference between this enzyme and cruzain lies in the S2 subsite, where cruzain contains a glutamate residue at the base of the pocket, whereas TbrCATL contains an alanine residue [112].

#### 5.2.2. Biological Functions of TbrCATL

TbrCATL can be found in the lysosomes and is essential for the parasite survival [46,114,115]. In HAT, TbrCATL is also responsible for the parasite crossing the BBB, as it initiates the calcium signaling cascade by activating protease-activated receptors [116,117]. It was also demonstrated that these enzymes can help the parasite evade the mammalian immune system by changing the surface antigens during its life cycle and degrading IgG directed to the variant surface glycoprotein (VSG) [118].

#### 5.2.3. TbrCATL as a Therapeutic Target

Analogous to the strategies employed for cruzain, considerable effort has been devoted to identifying selective TbrCATL inhibitors as a therapeutic approach for HAT [119,120,121]. However, the parasite exhibits remarkable biological resilience. Consequently, several studies suggest that near-complete inhibition of the enzyme is required to achieve potent trypanocidal activity [56,116]. These findings underscore the need for pharmacological agents that not only are selective for TbrCATL but also capable of suppressing its activity to an extent sufficient to impair parasite survival.

### 5.3. Cathepsin L-like and Cathepsin B-like in Leishmania *spp.*

CPs have critical roles not only in parasites’ life cycles but also in their pathogenesis [40,47,51]. They can be found in different stages of *Leishmania* spp. and are fundamental for parasite nutrition, growth and replication, being in major concentrations in the lysosomal and flagellar pocket regions [79,122,123]. CPA, CPB and CPC are the major known CPs in *Leishmania* spp. CPB plays a crucial role in macrophage infection, differentiation and autophagy processes, and contributes to the modulation of the host immune response [79,124]. CPC is found in all stages of the parasite life cycle, but its knockout does not stop the infection [124,125,126]. To the best of our knowledge, no recent CPA inhibitors have been reported. Therefore, CPB currently represents the more promising therapeutic target for the treatment of leishmaniasis [40,127].

## 6. Evaluation Criteria for CP Inhibitors in Antitrypanosomatid Drug Discovery

The discovery and development of CP inhibitors as potential drugs against trypanosomatids infections usually follows the conventional drug discovery pipeline, beginning with target identification, followed by hit optimization [128,129].

The biochemical characterization of CP inhibitors is a critical step for validating their potential as drug candidates. There are several parameters to evaluate the inhibitor activity, with the half-maximal inhibitory concentration (IC_50_) and the inhibition constant (*K_i_*) being the most frequently used [130]. The IC_50_ value is determined from a concentration–response curve and represents the concentration of an inhibitor required to reduce enzyme activity by 50%. However, because IC_50_ is influenced by experimental conditions, including enzyme and inhibitor concentrations, it is not an intrinsic measure of inhibitor potency. Consequently, it is unreliable to compare the IC_50_ values obtained in different experiments, even if the laboratory conditions are very similar [130]. A compound is usually considered a hit when it exhibits an IC_50_ value below 1 µM (or 1 µg/mL) against the target enzyme [129]. On the other hand, the *K_i_* value is the dissociation constant of the enzyme–inhibitor complex and is an intrinsic parameter that reflects both the affinity of the inhibitor for the enzyme and its mechanism of inhibition. Thereby, *K_i_* provides more reliable criteria for comparing potency across different studies [130]. In general, compounds with *K_i_* values in the low nanomolar range are regarded as highly potent inhibitors [131,132,133]. For both IC_50_ and *K_i_*, lower values indicate greater inhibitory potency.

Additional kinetic parameters can be used to characterize inhibitors, depending on their mechanism of action. The second-order rate constant (*k_2nd_*) is the preferred parameter for irreversible inhibitors. This constant is usually determined by the ratio of the covalent enzyme inactivation rate constant (*k_inact_*) to the inhibitor dissociation constant (*K_i_*), which expresses the affinity of the inhibitor for the enzyme [120]. However, other methods are available, for example, *k_2nd_* can be determined directly from the dependence of the observed inactivation rate constant (*k_obs_*) on inhibitor concentration, using the appropriate kinetic model [127]. A high *k_2nd_* indicates the presence of an efficient irreversible inhibitor, usually because it either binds tightly, reacts rapidly, or both. On the other hand, a low *k_2nd_* indicates poor overall efficiency, even if one of the two individual parameters (*k_inac_* or *K_i_*) is favorable [130,134,135].

Although these parameters are fundamental for characterizing compounds as CP inhibitors, high enzymatic potency alone is insufficient to identify a promise hit compound [129]. In antiparasitic drug discovery, biochemical activity must be complemented by cellular efficacy, which is typically assessed relative to reference drugs such as benznidazole [128,136]. The half-maximal effective concentration (EC_50_), also referred to as IC_50_, determined from concentration–response curves, is a key parameter for evaluating drug candidates, as it represents the concentration needed to produce 50% trypanocidal or leishmanicidal activity or 50% inhibition of parasite growth [129].

Since trypanosomatids undergo multiple developmental stages during their life cycle, compounds should be evaluated against the clinically relevant forms present in the human host, such as amastigotes of *T. cruzi* and *Leishmania* spp., rather than relying solely on activity against extracellular forms [128,136]. This approach provides a more physiologically relevant assessment of antiparasitic efficacy and improves the predictive value of pre-clinical screening. The expected antiparasitic potency of a hit compound varies according to the target parasite. For *T. cruzi*, compounds are generally considered active when they exhibit an EC_50_ below 1 µg/mL (or an IC_50_ < 10 µM toward amastigotes). For *T.* *brucei rhodesiense*, the recommended threshold for hit compounds is an EC_50_ below 0.2 µg/mL, whereas for *T. brucei brucei*, compounds are considered to have reasonable activity when they display an IC_50_ below 0.5 µM. For *Leishmania* spp., hit compounds should demonstrate activity against intracellular amastigote stage within macrophages, with EC_50_ values between 1 and 2 µg/mL for *Leishmania donovani* and *Leishmania infantum* [129,136]. There is also indication that the IC_50_ against the amastigote form of *L. donovani* should be less than 10 µM [136].

Assessment of cytotoxicity against mammalian cells is an essential part of the antiparasitic drug development. Cytotoxicity is typically expressed as half-maximal cytotoxic concentration (CC_50_), which is compared to the antiparasitic EC_50_ to calculate the selectivity index (SI = CC_50_/EC_50_). The SI provides a measure of the therapeutic window by reflecting the compound’s selectivity for the parasite over host cells. In general, a compound is considered a hit when it exhibits an SI greater than 10, although more stringent criteria are often applied [128]. For *T. b. rhodesiense*, a SI > 100 is generally recommended, whereas values > 50 for *T. cruzi* and >20 for *Leishmania* spp. have been proposed as minimum thresholds for promising compounds [129]. Other authors advocate an SI > 100 for both Chagas disease and leishmaniasis to ensure an adequate safety margin [136].

The SI can also be used to evaluate the specificity of enzyme inhibitors by comparing their activity against the parasite CPs and the corresponding human homolog. In this context, a selectivity of at least 10-fold in favor of the parasite enzyme is generally considered desirable for a promising lead compound [129].

To summarize, in addition to enzymatic and biological activity, favorable chemical structure and pharmacokinetic properties are essential for a hit to be considered a lead compound [129].

## 7. Highlights in Cysteine Protease Inhibitors Against Trypanosomiasis and Leishmaniasis

### 7.1. Reference Inhibitors

**Figure 6:**
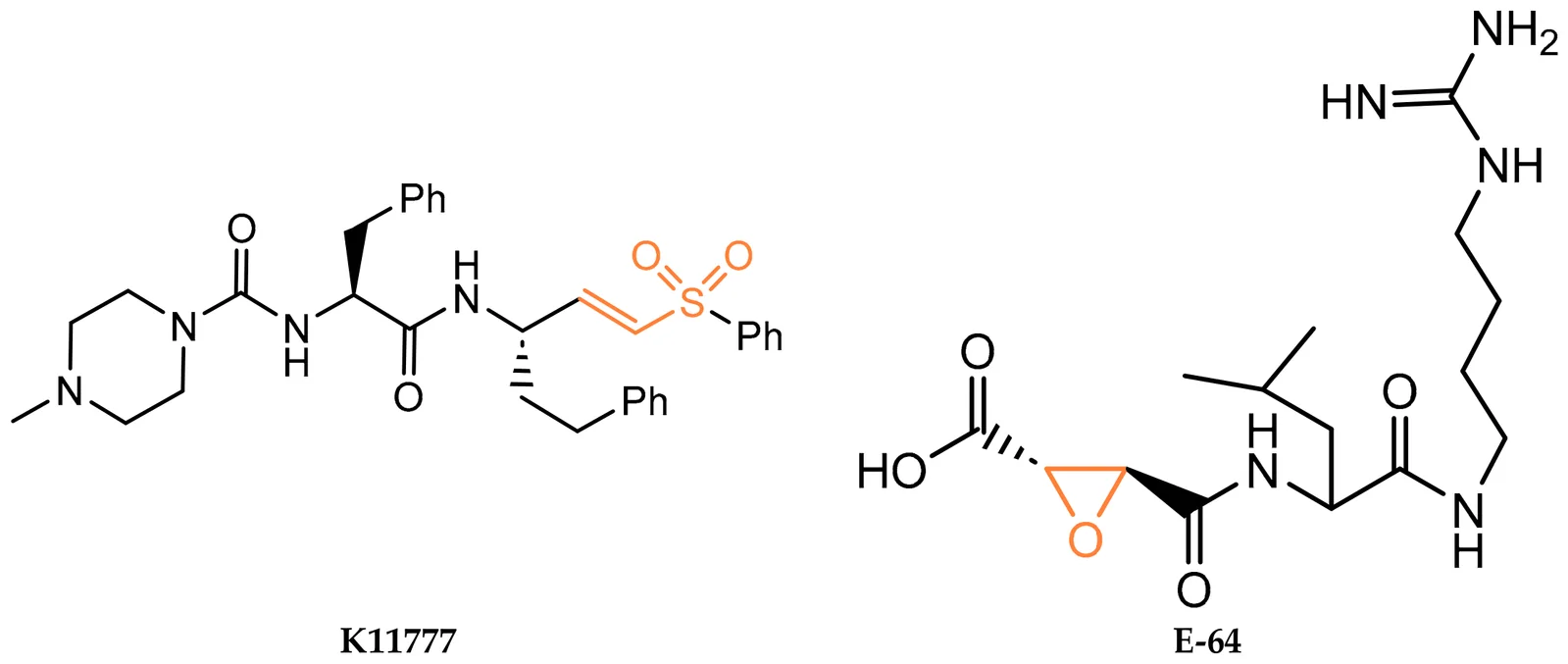
Compound K11777 and compound E-64. The structural motifs responsible for the irreversible inhibition of CPs are highlighted in orange (vinyl sulfone group for K11777 and epoxide moiety for E-64).

Given the well-established role of CPs in parasite biology and virulence, the development of CP inhibitors has been widely pursued. K11777 (Figure 6), a phenyl vinyl sulfone, was among the first CP inhibitors to reach the pre-clinical phase [137,138]. This compound has effects in both *T. cruzi* and *T. brucei*, and in *Leishmania* spp. cell cultures, altering the parasites’ growth [76,139]. Unfortunately, K11777 never reached the market, due to problems with tolerability, which are usually present in compounds with irreversible mechanisms of inhibition. In this reference derivative, the vinyl sulfone group (marked in orange in Figure 6) is responsible for the covalent bond with the catalytic cysteine thiolate residue of CPs [74,75,76]. As for E-64, another well-known irreversible CP inhibitor, originally obtained from the fungus *Aspergillus japonicus*, and now also frequently used as a reference compound for evaluating the enzymatic activity of newly discovered CP inhibitors, the epoxide moiety (also highlighted in orange in Figure 6), which undergoes nucleophilic ring opening by the catalytic cysteine, is responsible for the inactivation of the active site of CPs [120,140,141,142]. However, its high potency is offset by the lack of selectivity, a common limitation of this class of inhibitors [140,143].

### 7.2. Peptidic-Related Compounds

Peptidic-related compounds are a common strategy used to develop CP inhibitors. They can act by a reversible or an irreversible mechanism and comprise a series of compounds designed from amino acid scaffolds [80,144,145].

#### 7.2.1. Peptidic Compounds Containing Urea Linkages

Peptidic compounds containing Michael acceptor moieties are associated with an irreversible mechanism of inhibition, due to their ability to react with the catalytic thiol group of the CP and form a covalent bond [120,145]. Urea derivatives bearing Michael acceptors groups have exhibited nanomolar *K_i_* values against TbrCATL and promising EC_50_ values against *T. brucei*. Notably, some of these compounds displayed *k_2nd_* higher than that of K11777, revealing an extremely rapid and efficient enzyme inactivation. Compounds **1** and **2** are examples of this class of inhibitors [120].

**Figure 7:**
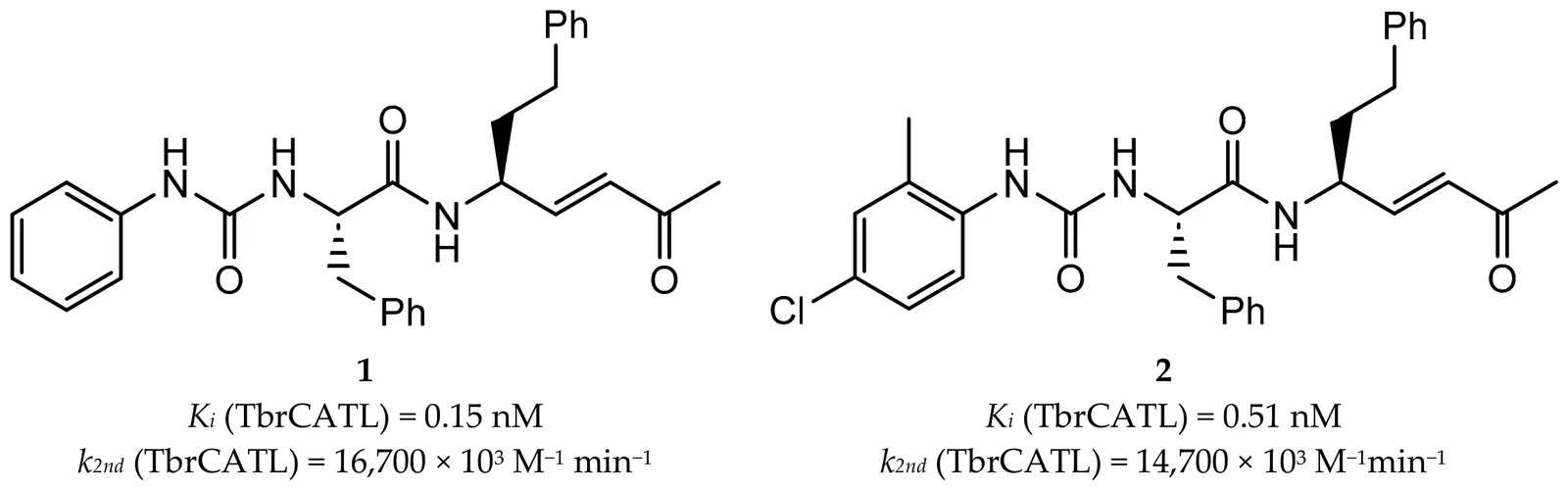
Urea-based TbrCATL inhibitors.

Compound **1** (Figure 7) emerged as the most potent derivative against rhodesain, with a *K_i_* = 0.15 nM and *k_2nd_* value of 16,700 × 10^3^ M^−1^ min^−1^. However, despite exhibiting lower potency toward human cathepsin L (hCatL) (*k_2nd_* = 5920 × 10^3^ M^−1^ min^−1^), this compound displayed limited activity against *T. brucei* (EC_50_ = 19.79 μM), probably due to its poor cellular permeability [120]. On the other hand, compound **2** has a 4-chloro-2-methyl-phenyl group, which is responsible for the lipophilic interactions with the S3 pocket of the rhodesain active site making it the most promising urea-based derivative. Indeed, compound **2** exhibited a *K_i_* value of 0.51 nM with a *k_2nd_* of 14,700 × 10^3^ M^−1^min^−1^, while maintaining trypanocidal activity (EC_50_ = 1.65 μM) [120]. Therefore, even though these compounds act through an irreversible mechanism, they still represent a pivotal advancement in the development of highly selective therapeutic agents against HAT. However, given that compound **2** also potently inhibits hCatL (*k_2nd_* = 3110 × 10^3^ M^−1^ min^−1^), further toxicity studies are required to fully characterize the safety profile of both compounds **1** and **2** [120].

These findings raise important questions regarding whether selective irreversible inhibitors may induce significant toxicity as an inherent consequence of their covalent mechanism of action, thereby requiring further safety optimization, or whether off-target toxicity can be mitigated through the development of highly selective inhibitors, making target selectivity the primary therapeutic strategy to pursuit [120,138]. However, for the time being, current research has increasingly focused on the development of covalent but reversible compounds [131,132,146].

#### 7.2.2. Peptidic Compounds Bearing the Vinyl Sulfone Scaffold

**Figure 8:**
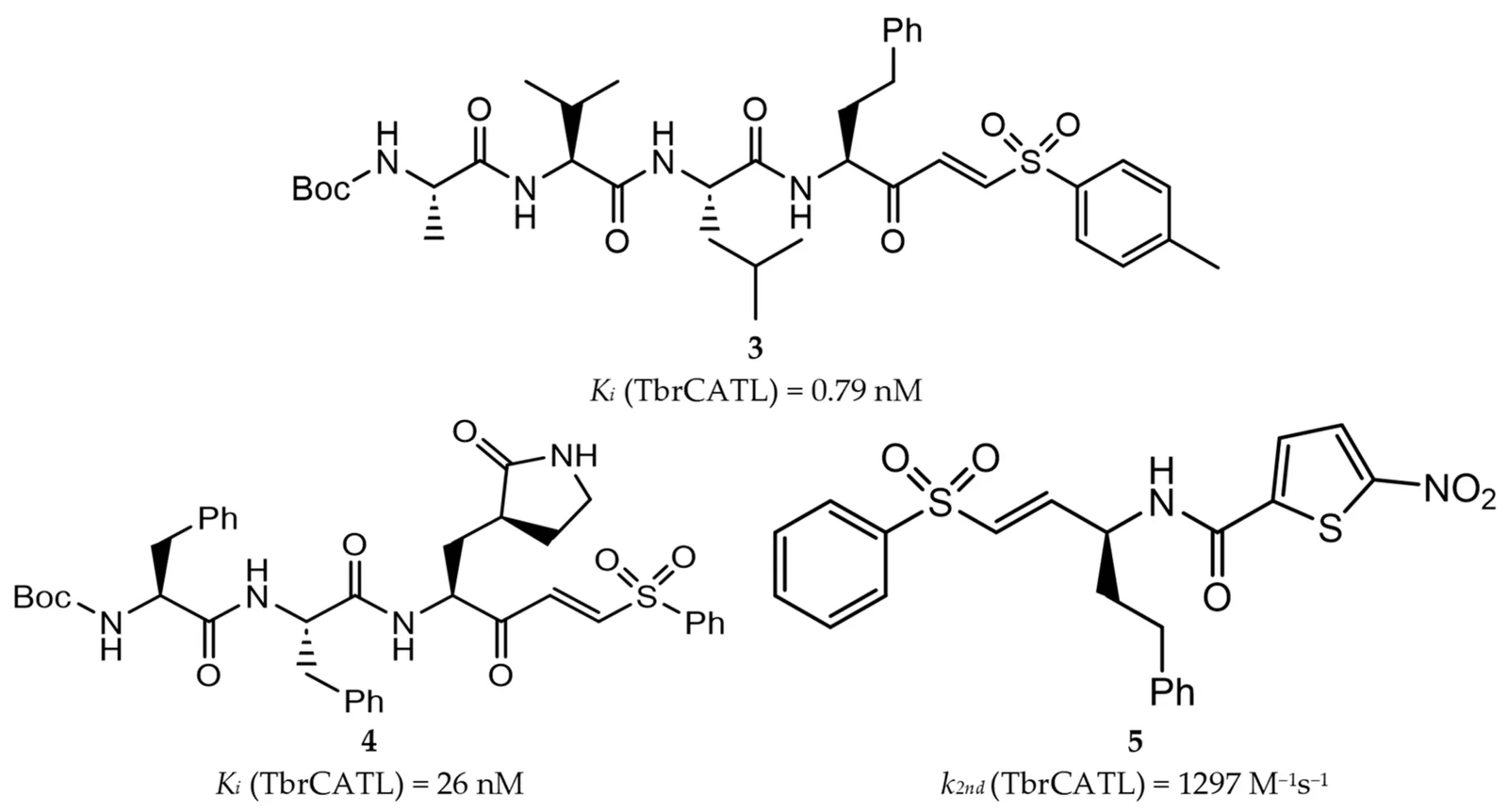

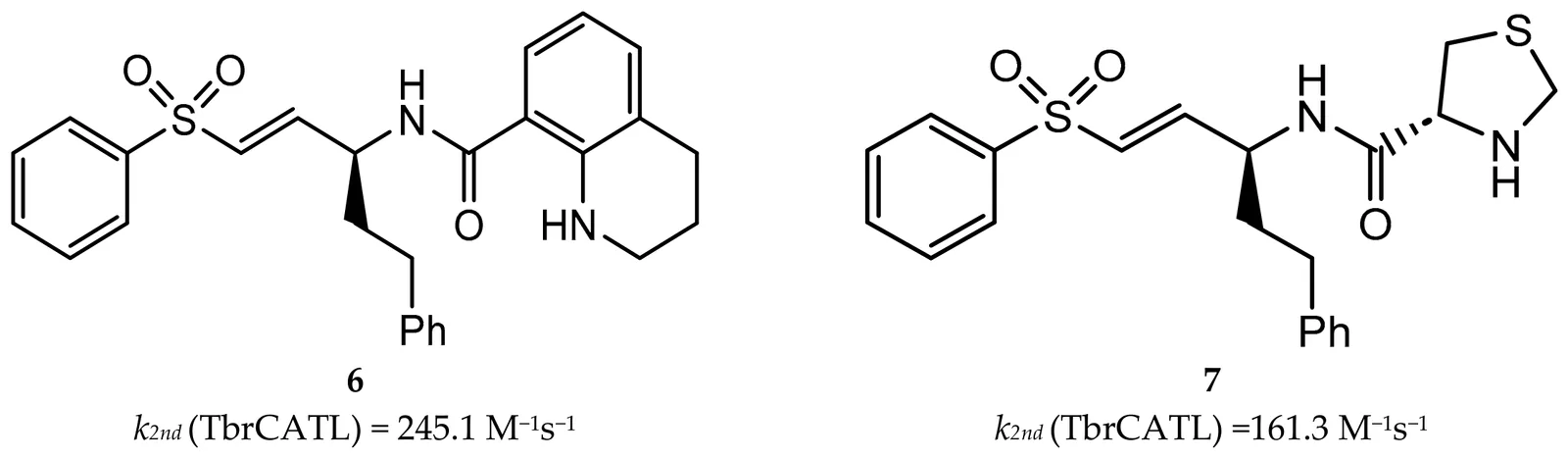
Peptidic bearing the vinyl sulfone scaffold.

As discussed for K11777, the vinyl sulfone moiety remains an important irreversible warhead in the development of CP inhibitors. This scaffold has recently contributed to the discovery of compounds **3** to **7** (Figure 8), which are peptidic inhibitors of rhodesain. Compounds **3** and **4** exhibited *K_i_* values in the nanomolar range, with compound **3** having the highest potency against rhodesain (*K_i_* of 0.79 nM) and a *K_i_* value of 86 nM toward hCatL, resulting in an SI greater than 100 for the parasite enzyme. Despite its lower potency, compound **4** still exhibits more than 10-fold selectivity toward the parasite enzyme. This high degree of selectivity provides a strong rationale for further investigation of its trypanocidal activity [80].

Compound **5** exhibits a *k_2nd_* of 1297 M^−1^s^−1^ against rhodesain, revealing its affinity for the active site of the enzyme and that its inactivation happens relatively quickly. This compound was able to inhibit TbrCATL at 100% at 10 μM and demonstrated an EC_50_ of 0.69 μM against *T. brucei*, presenting an SI of 18.01, compared to its cytotoxicity against Hep G2 cells (CC_50_ = 12.43 μM) [119].

Zhang and colleagues were also able to identify compounds **6** and **7**, both capable of almost complete inhibition of TbrCATL at 10 μM. Notably, compound **6** demonstrated an SI greater than 25, while maintaining a moderate inactivation efficiency (*k_2nd_* = 245.1 M^−1^s^−1^) [119]. These findings suggest that despite the presence of the vinyl sulfone scaffold, the nitrothiophene group might also be important for rhodesain inhibition in these compounds. However, further investigations are needed to ensure that there is no mammalian toxicity associated with the nitro group and to improve the SI so that it reaches the expected value (>100) against *T. brucei rhodesiense* [129,147,148].

#### 7.2.3. Peptidic Compounds Bearing the Nitrostyrene or the Nitro-Benzenic Group

**Figure 9:**
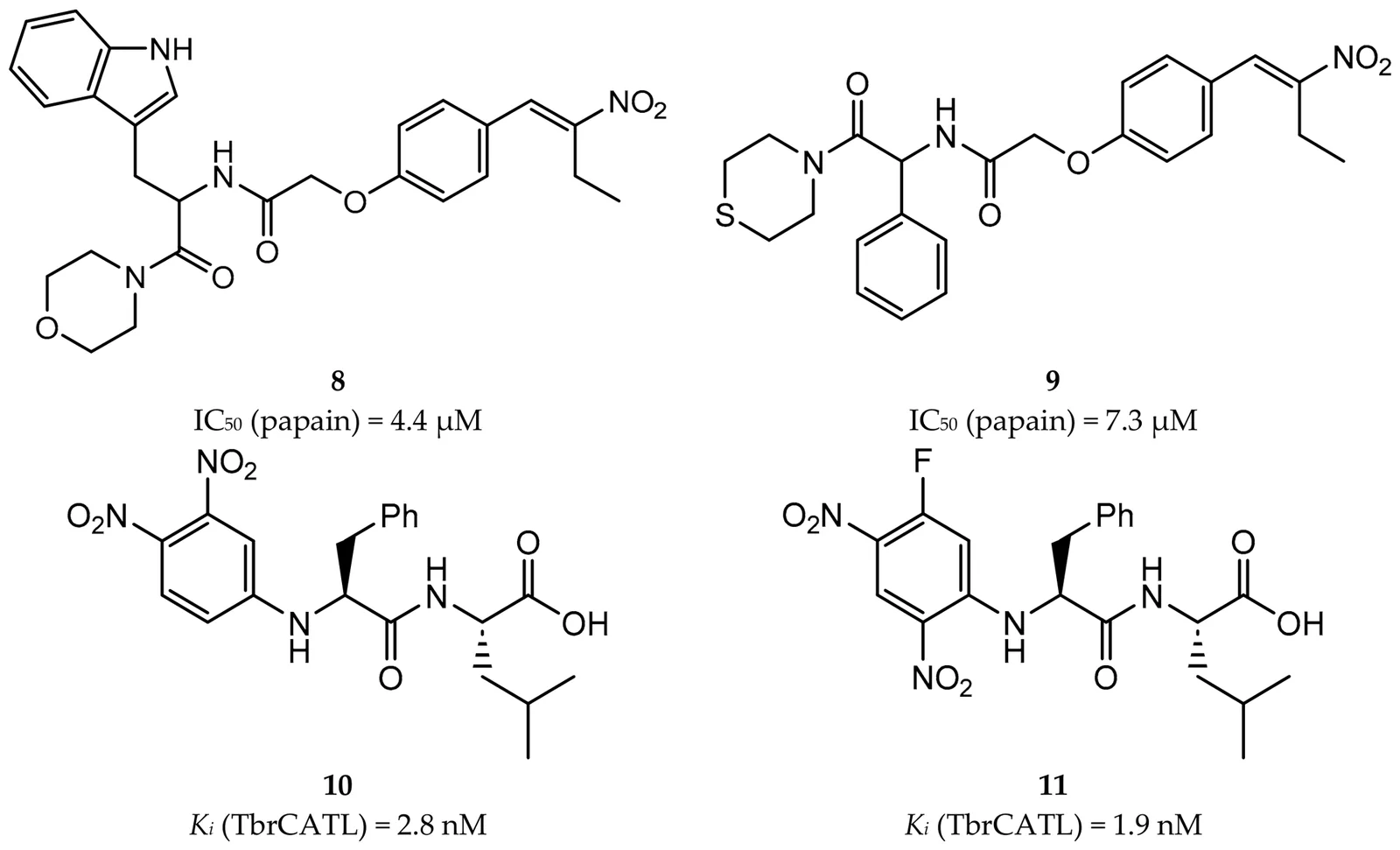
Peptidic compounds bearing the nitrostyrene (**8** and **9**) or the nitro-benzenic group (**10** and **11**).

Peptidic compounds containing β-nitrostyrene moieties represent another recent class of CPs inhibitors, with several derivatives showing activity against both the promastigote and amastigote forms of *L. donovani*. Among this class, compounds **8** and **9** (Figure 9) were evaluated against papain and exhibited IC_50_ values of 4.4 µM and 7.3 µM, respectively. Molecular studies revealed that these compounds interact with *L. mexicana* CPB through a combination of hydrophilic and hydrophobic non-covalent interactions [145]. In addition, compound **9** exhibited an IC_50_ of 0.64 µM against amastigotes form, a lower value compared with its enzymatic inhibition, suggesting that this compound may also affect additional cellular pathways. Unfortunately, cytotoxicity was observed in THP-1 cells at concentrations below 3 µM, highlighting the need for further optimization to minimize potential side effects. Furthermore, despite the presence of the β-nitrostyrene group, the authors did not report any studies or specific assays to exclude the possibility that these derivatives are pan-assay interference compounds (PAINS), which limits the interpretation of their potential as CP inhibitors [145].

Zimmer and colleagues also studied peptidic inhibitors and identified compounds **10** and **11**, which demonstrated potent inhibitory activity against TbrCATL. These compounds exhibited *K_i_* values of approximately 2.8 nM and 1.9 nM, respectively [144]. Moreover, the compounds bearing the nitro-benzenic group were reported as extremely selective toward the parasite enzyme, exhibiting an SI above 1000, calculated based on the ratio *K_i_*(TbrCATL)/*K_i_*(hCatB or hCatL). However, limited cellular permeability is a common challenge associated with peptide-based compounds [144,145]. For this reason, further studies were made leading to the conclusion that ester-based derivatives are more lipophilic and improved cellular uptake, even though carboxylic acids are more active against the enzyme [144]. These findings suggest that replacing the carboxylic acid group of a compound by an ester linkage could represent a promising strategy for a prodrug development. The underlying strategy involves the design of an ester-based prodrug that, following intracellular uptake, undergoes enzymatic hydrolysis to release the corresponding carboxylic acid, thereby yielding a more potent inhibitor [144,149].

#### 7.2.4. Dipeptidic Compounds Bearing the Nitrile Group

Developing a single inhibitor capable of targeting multiple kinetoplastid parasites is particularly important in the context of NTDs, as a broad-spectrum drug candidate may be more economically viable and more likely to attract funding. In this regard, the dipeptidyl nitriles class represents a promising starting point, as these compounds can exhibit activity against a broad range of papain-family CPs, making this chemical class particularly relevant for the current research objectives [82,121,149,150].

**Figure 10:**
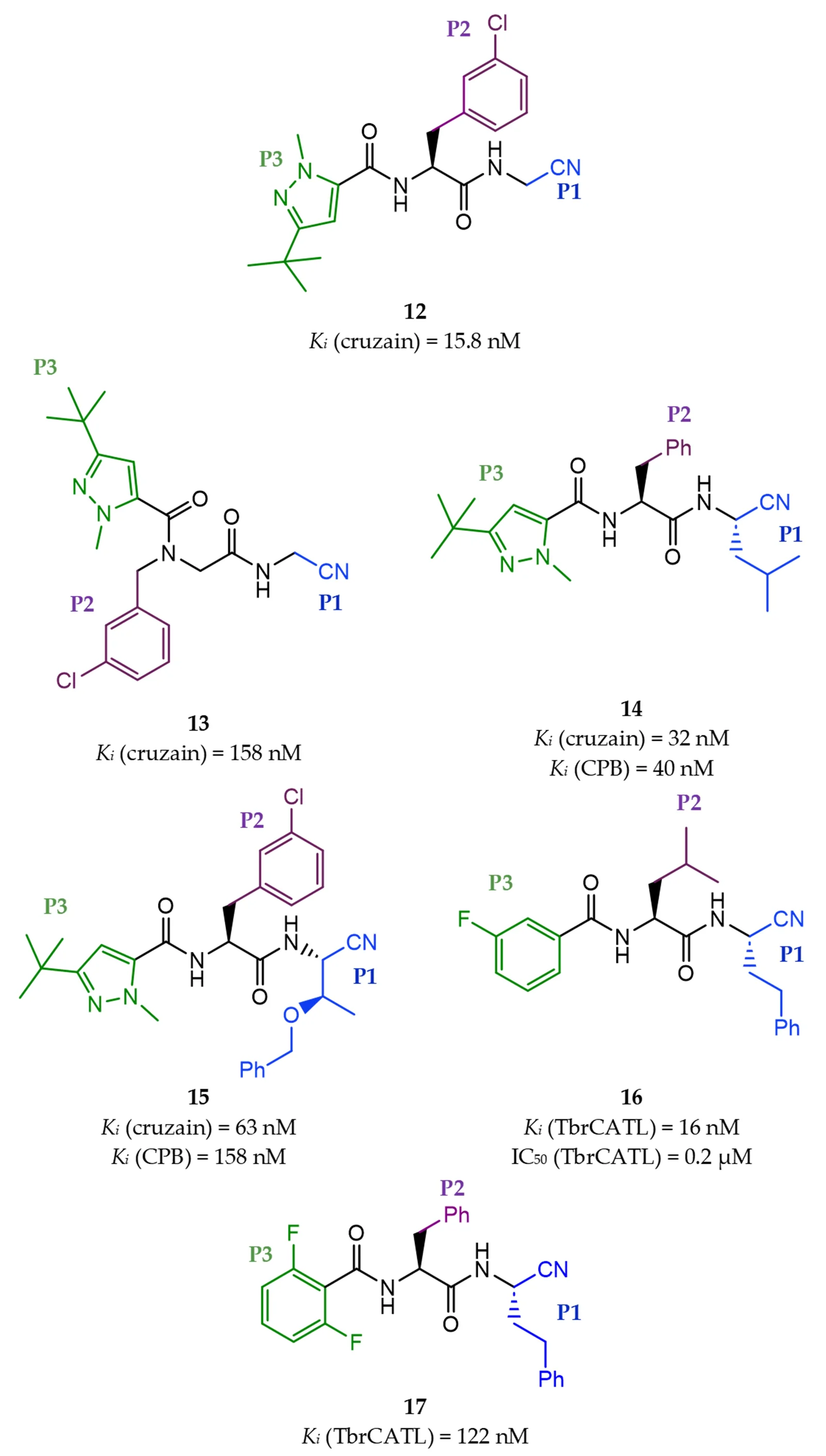
Dipeptidic compounds bearing the nitrile group.

Using the dipeptidyl nitrile scaffold, compound **12** (Figure 10) was identified as the most potent derivative of this class against cruzain, exhibiting a *K_i_* value of 15.8 nM (p*K_i_* = 7.8). This potency was conferred by the bond of the 1-methyl, 3-t-butylpyrazol-5-yl group (P3) to the S3 pocket of the enzyme, while S2 was occupied by 3-chlorophenyl (P2) and S1 by the nitrile moiety (P1). However, enzyme inhibition alone does not necessarily translate into enhanced trypanocidal activity [151].

Compound **13**, derived from the lead molecule Neq0415, is a potent inhibitor of cruzain (p*K_i_* = 6.8) and shares identical moieties with compound **12**. However, its potency is 10-fold lower, highlighting the importance of the molecular scaffold in the development of CPs inhibitors. Moreover, even though compound **13** represents a promising starting point for the development of selective inhibitors of cruzain and displays significantly lower potency against human cathepsin B (hCatB) (p*K_i_* = 5.1), it is capable of inhibiting more than 95% of the activity of other human CPs at 10 µM, like hCatL, possibly leading to toxicity issues [152].

On the other hand, compound **14** demonstrated a balanced activity profile, being a potent inhibitor of both cruzain and *L. mexicana* CPB (Table 1) [153]. Although its highest potency was observed against hCatL (p*K_i_* = 8.3), the compound maintained a favorable SI of 24 when comparing its trypanocidal activity with cytotoxicity toward the host cell (CC_50_ = 97.9 µM). Furthermore, its activity against *T. cruzi* amastigotes was comparable to that of benznidazole. Similarly, compound **15** was able to inhibit both cruzain and CPB, despite exhibiting a more elevated *K_i_*, probably not associated with the halo substitution in the P2 site but rather with its P1 moiety. Moreover, even though both compounds inhibit human cathepsins, they still display a favorable cytotoxicity profile [153].

Within the same class of compounds, derivatives **16** and **17** exhibited micromolar antitrypanosomal activity in addition to showing nanomolar potency against rhodesain, with *K_i_* values of 16 nM and 122 nM, respectively. These compounds demonstrated trypanocidal activity against *T. brucei brucei*, with EC_50_ values of 14.1 µM and 8.8 µM, respectively. Moreover, both compounds are predicted to cross the BBB and to possess oral bioavailability, indicating favorable drug-like properties, particularly for the treatment of *T. brucei* infections involving the central nervous system [121]. In subsequent studies, compound **16** exhibited an SI greater than 6.9 toward the parasite when comparing its EC_50_ against the parasite with its CC_50_ in HEK293 cells [154]. Although the SI between rhodesain and hCatL (*K_i_* = 34 nM) was only 2.12, the overall data indicates that compound **16** still exhibits a degree of selectivity toward the parasite. Consequently, this molecule, bearing a 3-fluorosubstitute in the phenyl ring of the P3 site and an isopropyl moiety at P2, represents a promising scaffold for the development of anti-HAT targeting TbrCATL [141,153,154].

#### 7.2.5. Peptidyl Enoates Compounds

**Figure 11:**
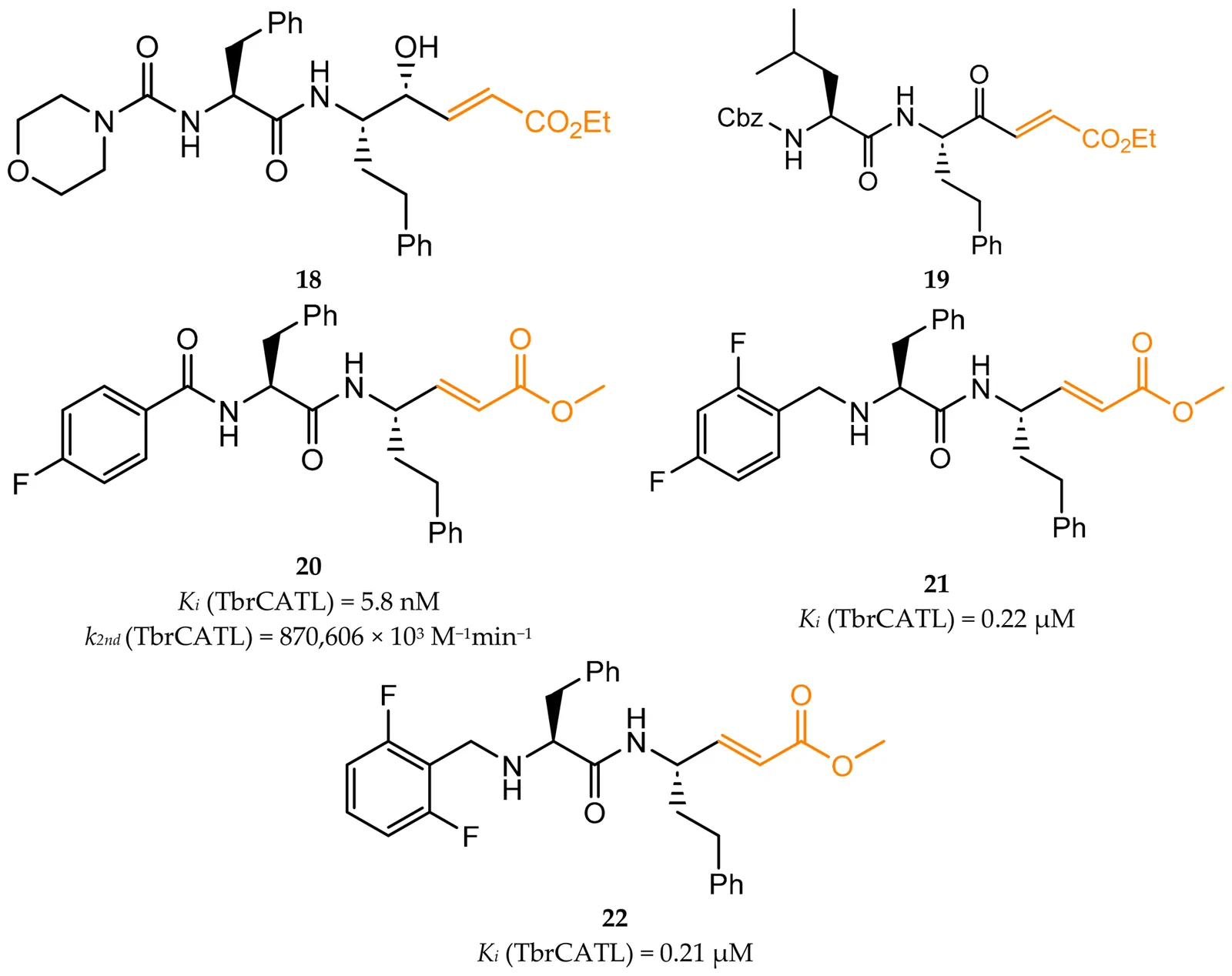
Peptidyl enoates as CP inhibitors. The electrophilic warhead of the compounds (methyl and ethyl vinyl ester group) is highlighted in orange.

Consistently, peptidyl enoates can also inhibit CPs (Table 2) [155]. In this class of molecules, Royo and collaborators found that compound **18** (Figure 11) inhibits 95% of cruzain (IC_50_ = 3.75 μM) and 99% of rhodesain (IC_50_ = 1.13 μM) at 20 μM. However, it is also able to inhibit hCatB (IC_50_ = 1.13 μM) and hCatL (IC_50_ = 2.45 μM) in almost 100% at 20 µM, revealing the lack of selectivity. Among all the tested compounds, none were able to selectively inhibit the assayed enzymes. For instance, compound **19** could inhibit cruzain, rhodesain, cathepsin B and cathepsin L at more than 98% at 20 μM. Besides its potency against the enzymes, compound **19** has antiprotozoal activity with an IC_50_ value of 7.14 μM against *T. cruzi*, 2.32 μM against *T. brucei rhodesiense*, and 0.09 μM against the axenic amastigote form of *L. donovani*. Moreover, in the cytotoxicity assay, cell viability remains superior to 50% even at 50 μM [155].

These findings indicate that compound **19** remains a viable scaffold for structural optimization against multiple NTDs, suggesting preferential selectivity toward the parasite, despite the simultaneous inhibition of host enzymes and irreversible mechanism, which is particularly pronounced toward rhodesain (*k_2nd_* = 116,200 M^−1^s^−1^) [155].

Compound **20** is also a peptidic enoate inhibitor that presents an electrophilic warhead (methyl vinyl ester group highlighted in orange in Figure 11) capable of binding to the catalytic site of TbrCATL. This compound is of particular interest given its high potency (*K_i_* = 5.8 nM and *k_2nd_* = 870,606 × 10^3^ M^−1^min^−1^) against this enzyme, despite also being active against hCatL (*K_i_* = 3.5 μM). However, it presents an EC_50_ value of 15.8 μM against *T. b. brucei*, indicating that further optimization is required before it can be considered a promising lead for the development of selective rhodesain inhibitors with trypanocidal activity. On the basis of this premise, compounds **21** and **22** were synthetized. However, both revealed to be less potent with *K_i_* of 0.22 μM and 0.21 μM, respectively. An important advantage of these compounds is the smaller EC_50_ (approximately 8.7 μM and 7 μM, respectively). Moreover, compounds **21** and **22** did not elicit significant cytotoxicity in HEK293 cells up to 70 μM and only inhibited hCatL at 38% and 26% at 100 μM, respectively [156].

These observations, aligned with the fact that a structurally related compound bearing a single fluorine atom at the para position of the phenyl ring presented a lower *K_i_* value, suggest that the carbonyl group might be crucial to potent interactions with the active site of the enzyme, probably by hydrogen bonding [156].

#### 7.2.6. Peptidic Compounds Bearing the Vinyl Ketone Group

**Figure 12:**
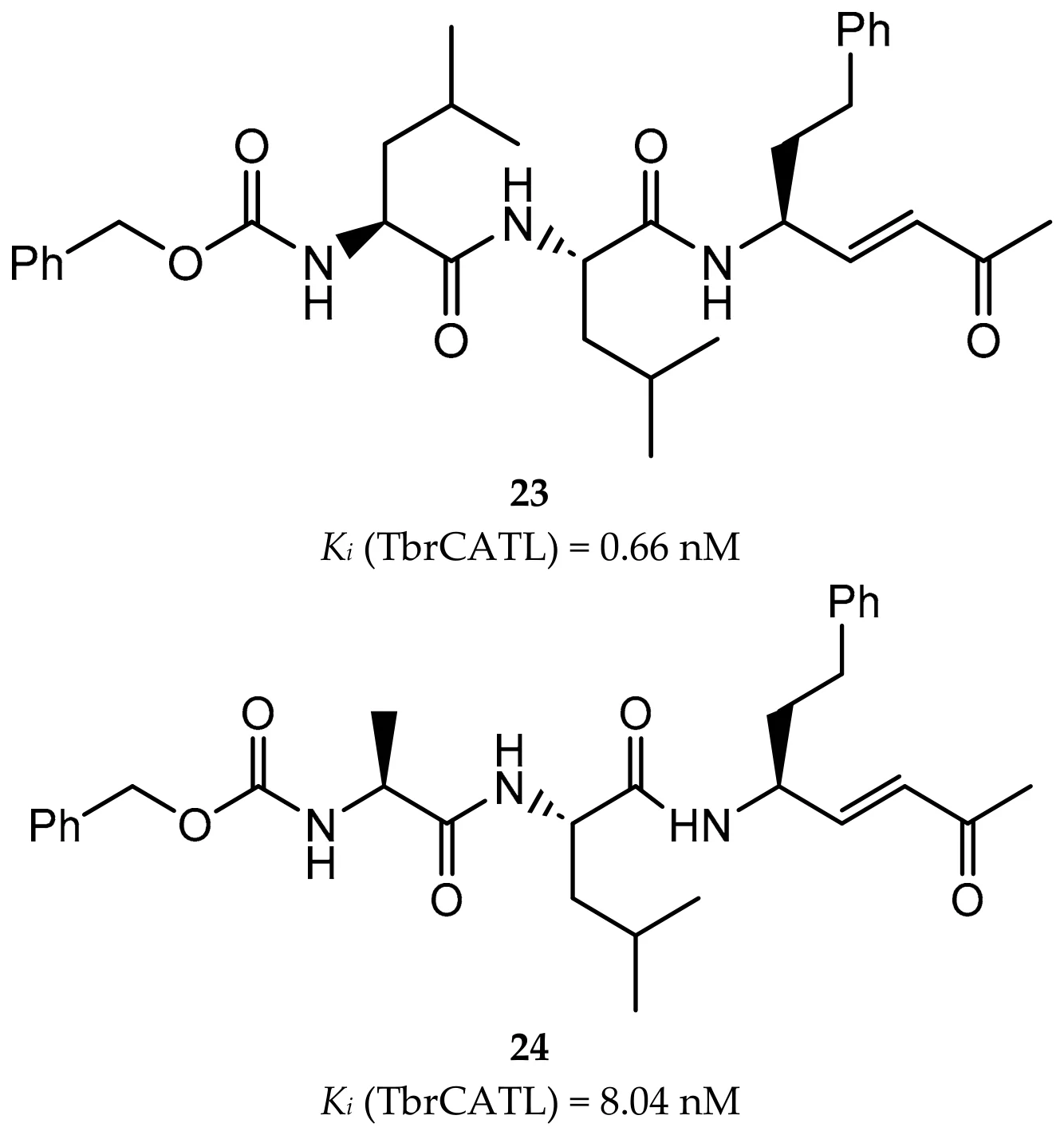
Peptidic compounds bearing the vinyl ketone group.

Even though less extensively studied than dipeptide compounds, tripeptide-based molecules have also demonstrated significant inhibitory capacity toward CPs (Table 3) [157,158,159]. Notable examples include the peptidic compounds bearing the vinyl ketone group, such as compound **23** (Figure 12), which has higher binding affinity for rhodesain, and compound **24**, which is also active toward the main CP of the causative agent of malaria. The design of these compounds enabled the investigation of the role of the residue occupying the S3 pocket of CPs: compound **24** has a small amino acid, Ala, while compound **23** has a bigger one, Leu. Conversely, compound **23** is the more active toward rhodesain, exhibiting a *K_i_* value in the nanomolar range (*K_i_* = 0.66 nM). Moreover, the compound is active against *T. brucei* (EC_50_ = 2.3 µM), demonstrating the importance of optimizing the P3 residue by increasing its molecular size to improve binding affinity within the S3 pocket of the enzyme [159].

#### 7.2.7. Peptidic Compounds Bearing the Vinyl-Pyridine Group

**Figure 13:**
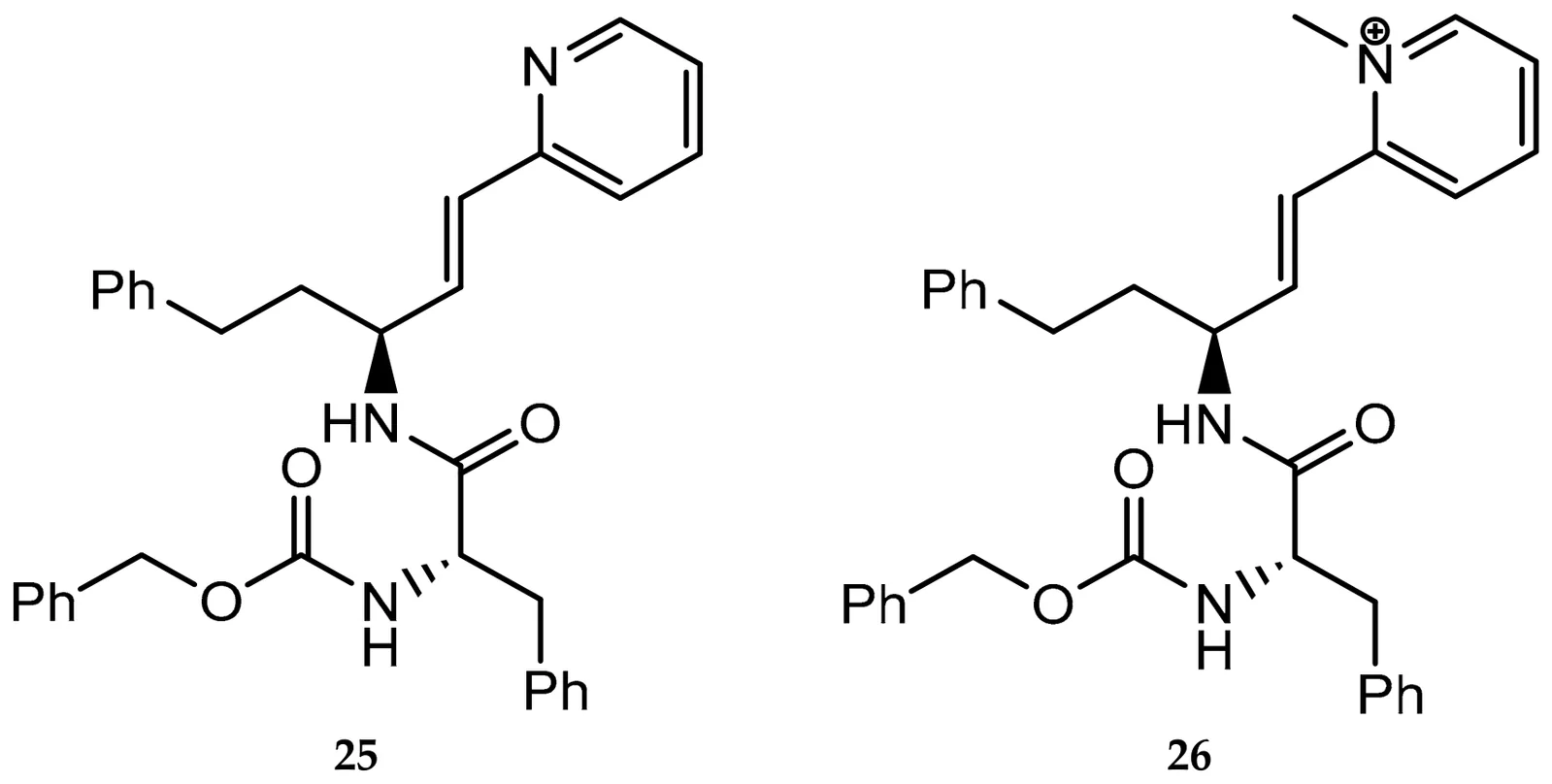
Peptidic compounds bearing the vinyl-pyridine group.

Using the vinyl-pyridine scaffold as a pharmacophore lead, Chenna and colleagues have identified different compounds as promising warheads in the design of potent and reversible time-dependent cruzain inhibitors [76]. The study reported *K_i_* values ranging between 0.1 and 0.4 μM, with compounds **25** and **26** (Figure 13 and Table 4) being the most noteworthy. These candidates target cruzain specifically, with *K_i_* values considerably lower for the parasite protease than for hCatL and hCatB. Moreover, they had antitrypanosomal activity, presenting EC_50_ values below 6 μM, and cytotoxicity assays revealed CC_50_ values greater than 100 μM in human cells. Consequently, these compounds exhibited an SI greater than 20, indicating a favorable therapeutic window and supporting their potential as candidates for the treatment of Chagas disease. Additionally, they demonstrated activity against *T. brucei brucei* with EC_50_ values ranging from 2.8 μM to 4 μM [76].

#### 7.2.8. Other Peptidic Compounds

**Figure 14:**
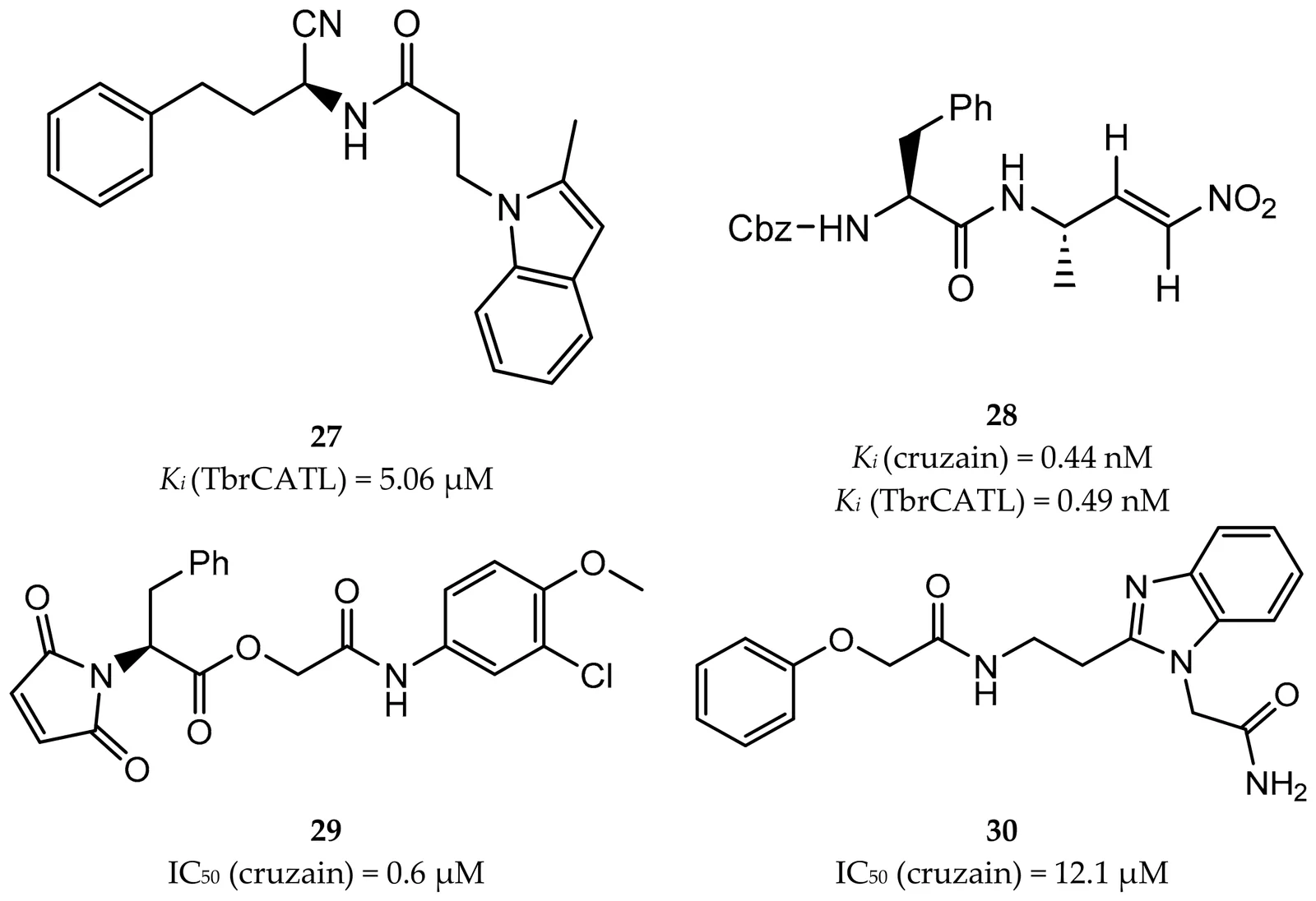
Other peptidic compounds.

Related to the peptidic structure, Di Chio and colleagues designed compound **27** (Figure 14), a derivative containing an alanine residue capable of inhibiting both rhodesain (*K_i_* = 5.06 µM) and falcipain-2 (IC_50_ = 40.43 µM), being a possible lead not only against *T. brucei* but also against the causative agent of malaria. This example highlights the importance of further optimizing compounds in order to find new therapy approaches for multiple NTDs [160].

Similarly, a nitroalkene peptidic compound with E configuration also inhibited CPs. Indeed, compound **28** is very potent against cruzain (*K_i_* = 0.44 nM) and human cathepsins (*K_i_* = 11 nM and *K_i_* = 8 nM against hCatL and hCatB, respectively), being the proposed interaction of this compound with cruzain by a Michael addition mechanism [161,162]. The same compound was also active against rhodesain at a nanomolar potency (*K_i_* = 0.49 nM) and has an EC_50_ value of 5.64 µM against *T. brucei brucei*. In this case, the Michael addition mechanism proposed results in a reversible covalent inhibition, a mechanism thought to be associated with fewer toxicity effects. However, the compound was considered unstable in the medium, leading to an increase in the EC_50_ value, reinforcing the importance of stability studies [161].

Following a different strategy, reversible cruzain inhibitors containing cyclic imides were designed with the objective of finding potential drug candidates for the treatment of Chagas disease. To the best of our knowledge, compound **29** is the most potent compound of this class, exhibiting an IC_50_ value of 0.6 µM, acting as a competitive and selective inhibitor of the enzyme. The compound exhibited selectivity toward *T. cruzi* (SI = 67, when tested against HepG2 cells). Furthermore, it exhibited greater trypanocidal activity than benznidazole against the amastigote form of the parasite (IC_50_ = 1.0 µM compared to IC_50_ = 3 µM for benznidazole), therefore possibly introducing a new class of inhibitors of CPs from trypanosomatids [163]. Benzimidazoles represent another class of compounds known as inhibitors of CPs [164,165]. Pauli and colleagues identified compound **30** as a reversible cruzain inhibitor that reduces enzyme activity by more than 80% at 100 μM, having an IC_50_ of approximately 12.1 μM [136]. The peptidic compound also exhibited potent antiparasitic activity against *T. cruzi* (IC_50_ = 6.8 μM) while showing low cytotoxicity toward HFF-1 cells (CC_50_ > 100 μM), resulting in an SI greater than 15. Moreover, in vivo, the compound was well tolerated at doses up to 150 mg/kg and retained moderate trypanocidal activity. Furthermore, the investigation conducted by this team extensively assessed the pharmacodynamics and pharmacokinetic properties of the tested compounds, illustrating their importance in drug development [136]. Compound **30**, for example, presents an amide group linked to the benzimidazole ring, which is responsible for its metabolic stability. This indicates that this hydrophilic group should be preserved during lead optimization, as it appears to be important for maintaining good pharmacodynamics and pharmacokinetic properties, which are crucial for the in vivo activity [165].

### 7.3. Other Classes of Chemical Compounds

There are different classes of chemical compounds well studied as CP inhibitors. Among them, compounds including triazole, thiosemicarbazones, naphthoquinone, isoxazole and aziridine groups are some of the most promising ones [77,81,127,131,132,146,166,167,168,169,170,171,172,173].

#### 7.3.1. Triazole-Based Compounds

**Figure 15:**
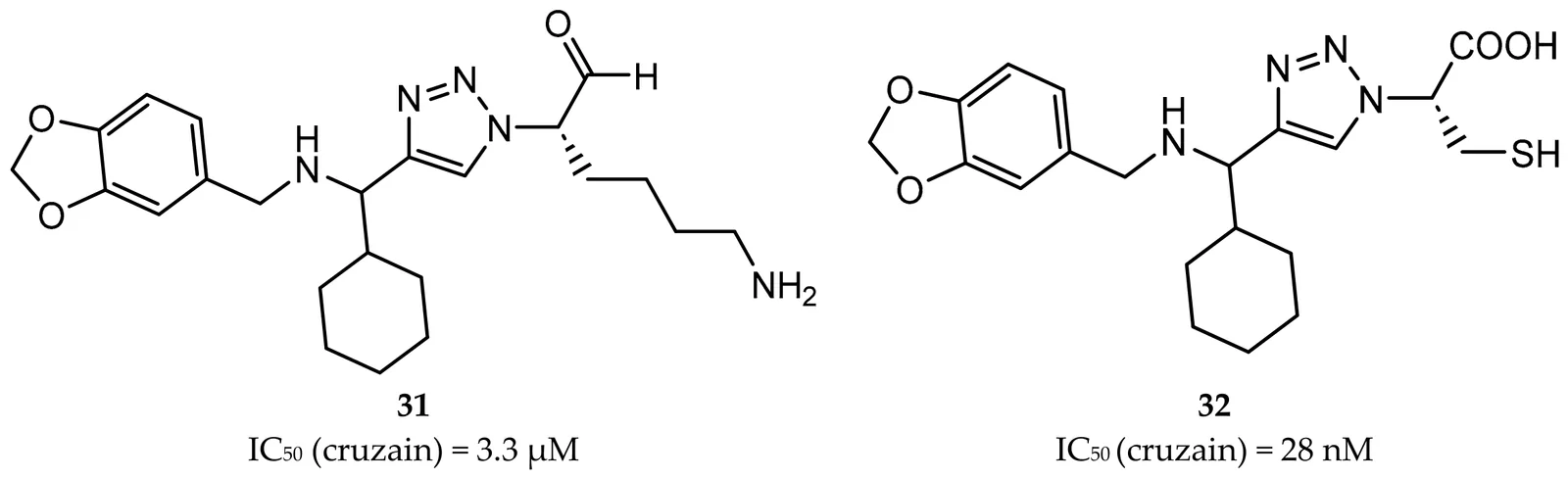
Triazole-based inhibitors of parasitic CP.

The triazole structure has been recently associated with cruzain inhibitors [81,132,174]. Cerutti and collaborators showed that compound **31** (Figure 15) has antitrypanosomal activity of 93% at 10 µM and inhibited cruzain with an IC_50_ value of 3.3 µM. More interestingly, this compound only inhibited the hCatL in 26%, indicating a high degree of selectivity for cruzain [81]. These findings demonstrate the potential of aldehyde warheads for targeting cruzain and highlight their promise as a strategy for the treatment of Chagas disease. This potential is further supported by the mechanistic studies performed by the authors, which suggested irreversible inhibition of cruzain, consistent with the known reactivity of the aldehyde group. Moreover, this derivative represents one of the few examples in which the potency of cruzain inhibition was directly associated with trypanocidal activity, with an IC_50_ of 3.2 µM against *T. cruzi* [81]. Moreover, in cytotoxicity assays, even at 50 µM, 80% of the cells maintained viability, making this compound an important scaffold for further studies [81].

Compound **32**, an even more potent compound with the triazole structure, was also identified by Cerutti and collaborators [132]. The only difference between this compound and compound **31** is the presence of a carboxylic acid warhead instead of an aldehyde. However, its reversible covalent inhibition of cruzain resulted in an increase in enzymatic potency (IC_50_ = 28 nM), with no inhibition of hCatL at 100 µM. On the other hand, the presence of the more acidic group led to a decrease in the potency against the parasite (IC_50_ = 6.67 µM), probably due to permeability limitations associated with its warhead [132].

#### 7.3.2. Thiosemicarbazones-Based Compounds

As mentioned, thiosemicarbazone compounds have recently gained more importance due to the discovery of their activity as potent CP inhibitors [77,131,146,167,168,169,170,171,172]. The most promising compounds of this class are described in Table 5.

**Figure 16:**
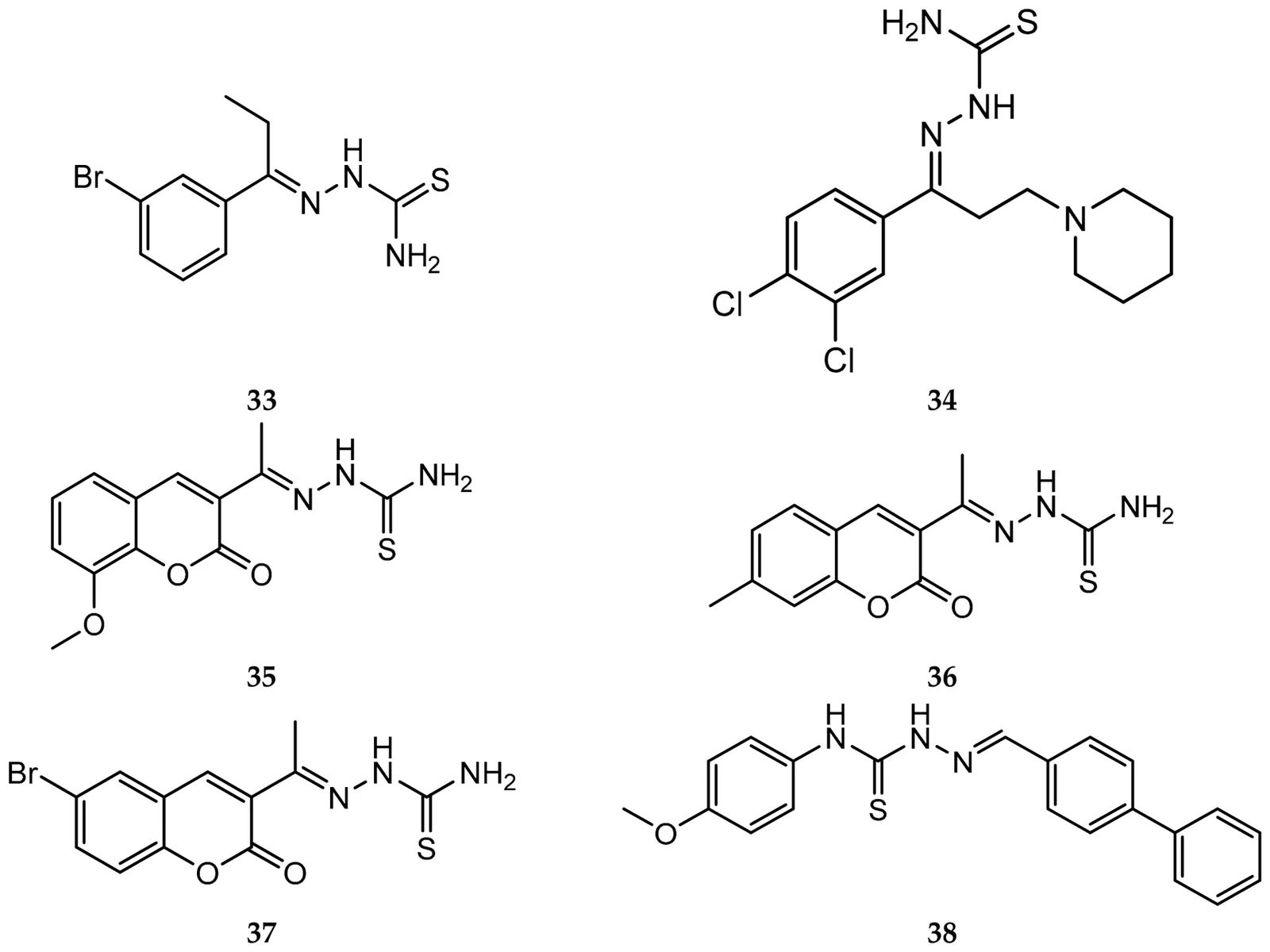
Thiosemicarbazone compounds as cruzain inhibitors.

Jasinski and collaborators used compound **33** (Figure 16) as a lead, further investigating thiosemicarbazones’ potential against parasitic CPs [146]. Compound **33** already exhibited IC_50_ around 170 nM against cruzain and 230 nM toward rhodesain. However, the efforts made to optimize the thiosemicarbazone moiety led to the design of derivative **34**, a double chlorine compound which exhibited significantly greater potency against cruzain. This new compound accommodates a piperidine group that bonds in the S3 cluster of cruzain, conferring it an IC_50_ of 2.86 nM, and complete inactivation of the enzyme. Moreover, according to the filter implemented on the SwissADME website, molecule **34** is not predicted to be a pan-assay interference compound (PAINS), an important characteristic for the reliability of the assays performed. However, the compound was not tested in *T. cruzi* cells, leaving the activity against the parasite to be determined [146,170,172].

On the other hand, Nunes and colleagues synthesized compound **35**, a coumarin-3-thiosemicarbazones derivative, which exhibited antiparasitic activity against intracellular *T. cruzi* (EC_50_ = 5.5 μM) and an IC_50_ of 14.4 μM against cruzain. However, despite greater potency against TbrCATL (IC_50_ = 2.0 μM), the compound is not active against *T. brucei* trypomastigotes at 10 μM [77].

Due to its enzymatic potency and low cytotoxicity (100% cell viability at 10 μM), the authors further optimized this scaffold using a bioisosteric replacement strategy, identifying compounds **36** and **37** as the most promising derivatives. Compound **36** exhibited great activity toward both proteases, achieving more than 90% inhibition of cruzain and TbrCATL at 50 μM. Nevertheless, compound **36** inhibited *T. b. brucei* trypomastigotes by only 23% at 10 μM and *T. cruzi* amastigotes at 52% at the same concentration. On the other hand, compound **37** showed great inhibition of both proteases, showing an IC_50_ value of 0.698 μM against TbrCATL and an IC_50_ value of 0.144 µM against cruzain. It also demonstrated improved antiparasitic activity, inhibiting *T. b. brucei* trypomastigotes by 71% at 50 μM and *T. cruzi* amastigotes by 53% at 10 μM. However, when evaluated in HEK-293 cells, the bromo derivative exhibited measurable cytotoxicity. Consequently, despite its predicted oral bioavailability and ability to cross the BBB, its low selectivity toward the parasite (SI ≈ 1.20) indicates that further optimization is required to improve its safety and selectivity profile [131].

Continuing the search for the therapeutic potential of thiosemicarbazones as antimicrobial agents, Meier and colleagues discovered that a derivative (compound **38**), predicted to have appropriate properties for oral absorption, inhibited cruzain with an IC_50_ value of 0.306 µM. This compound binds favorably to the active site of the enzyme, while presenting CC_50_ of 1.96 µM against *T. cruzi* trypomastigotes, a much lower concentration than that observed in the cytotoxicity assay (CC_50_ > 64 µM), conferring the compound an SI greater than 32 [171].

Therefore, the available evidence suggests that compounds containing thiosemicarbazone moieties can be potent inhibitors of both cruzain and rhodesain while exhibiting antitrypanosomal activity in the low micromolar range against *T. cruzi* and *T. brucei*, demonstrating the promising translation of their bioactivity into antiparasitic activity. However, further structural optimization is still needed to improve the SI of these compounds. Moreover, to the best of our knowledge, no recent advances have been made in the design of thiosemicarbazone compounds as inhibitors of CPs in *Leishmania* spp. However, these molecules exhibit leishmanicidal activity and, consequently, should be considered potential scaffolds for drug development [175,176,177,178].

#### 7.3.3. Aziridine-Based Compounds

**Figure 17:**
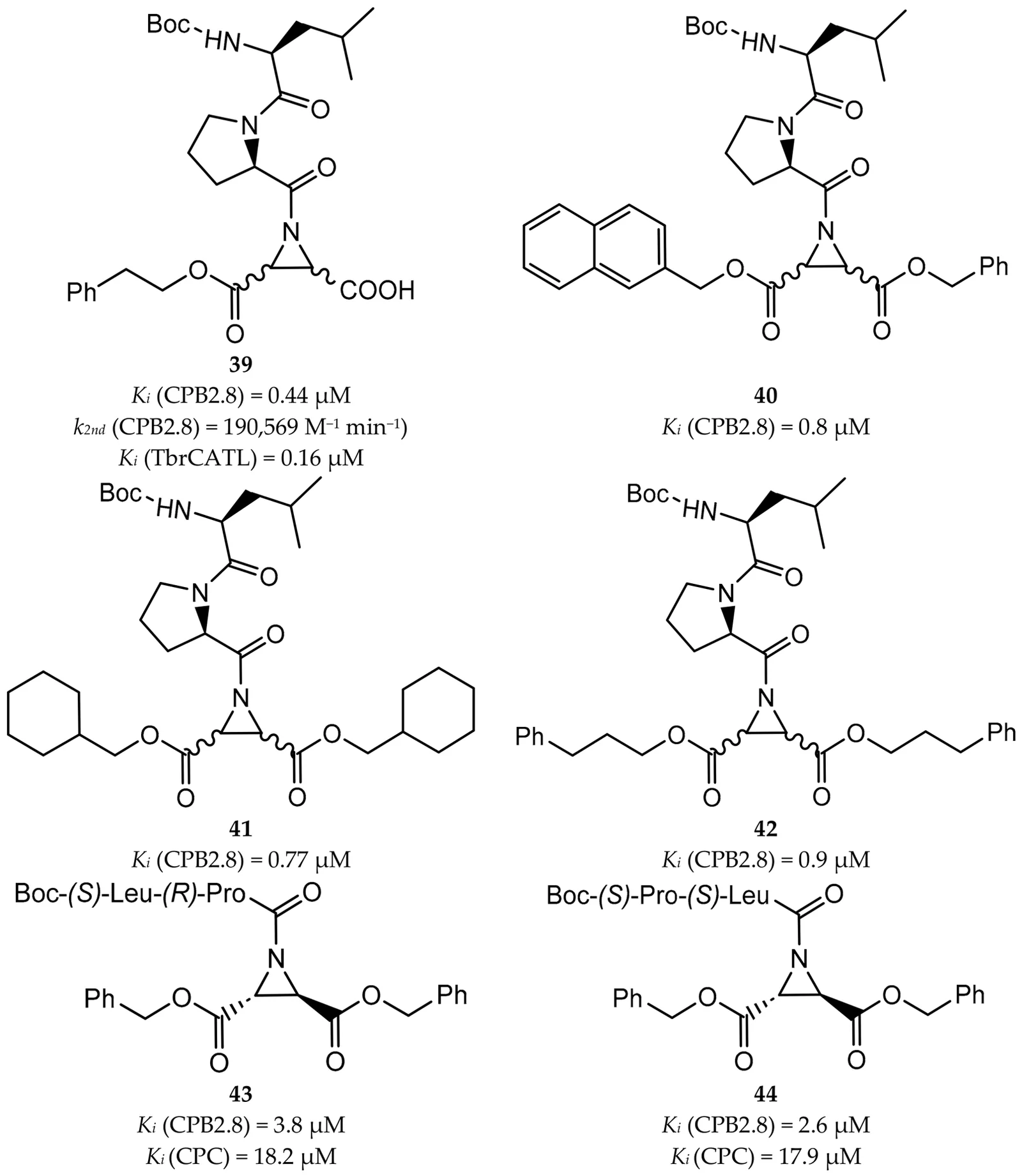
Aziridine compounds as CP inhibitors.

Aziridine compounds have already been proved to have in vitro leishmanicidal activity against the promastigote and amastigote forms of *Leishmania* spp. [48,124,127]. Furthermore, Schad and colleagues reported that these compounds displayed minimal toxicity toward host cells, making them favorable for the journey of finding selective inhibitors of CPs from *L. mexicana* [48]. Compound **39** (Figure 17) is a potent aziridine derivative with *K_i_* value of 0.44 µM (*k_2nd_* = 190,569 M^−1^ min^−1^) against CPB2.8. Moreover, this time-dependent inhibitor revealed some selectivity toward parasite enzymes since it could inhibit rhodesain (*K_i_* = 0.16 µM) but not the mammalian cathepsin L, making it not only a promising agent to treat leishmaniasis but also HAT [127]. However, this compound may present permeability limitations due to being an acidic compound, revealing the importance of balancing selectivity and potency with pharmacokinetic properties. On the other hand, compounds **40**, **41** and **42**, containing more lipophilic groups and an additional ester linkage, were also identified, with *K_i_* values ranging from 0.7 to 0.9 µM toward CPB2.8. Unfortunately, no more studies were made in order to investigate their potential in vivo [127].

In the same study, it was also suggested that the conformation of the aziridine ring does not particularly influence the linkage to CPB2.8 [127]. Moreover, they have synthetized compound **43**, which exhibits a *K_i_* value of 3.8 µM toward CPB2.8, with no inhibition of hCatL or hCatB [48]. This compound was also active against CPC from *L. major* (*K_i_* = 18.2 µM). Consequently, compound **43** was revealed to have potent leishmanicidal activity (IC_50_ = 2.3 µM toward the amastigote), with its preferential profile being emphasized by the lack of cytotoxicity (IC_50_ = 68 µM, against J774.1 cells) which confers it an SI superior to 29, a remarkable value that points for further research of this compound. Other aziridine compounds like compound **44** were also identified with activity against *L. mexicana* CPB2.8 and *L. major* CPC, revealing the possibility of a single inhibitor acting against more than one CP of different species of *Leishmania.* Moreover, despite the fact that compound **44** is also able to inhibit hCatL and hCatB, exhibiting *K_i_* values of 14.2 µM and 29.2 µM, respectively, it still shows no cytotoxicity toward J774.1 cells (IC_50_ > 100 µM). Unfortunately, no data are available regarding its activity against the amastigote [48].

#### 7.3.4. Other Oxygen-Containing Aromatic and Naphthalene-Based Derivatives

**Figure 18:**
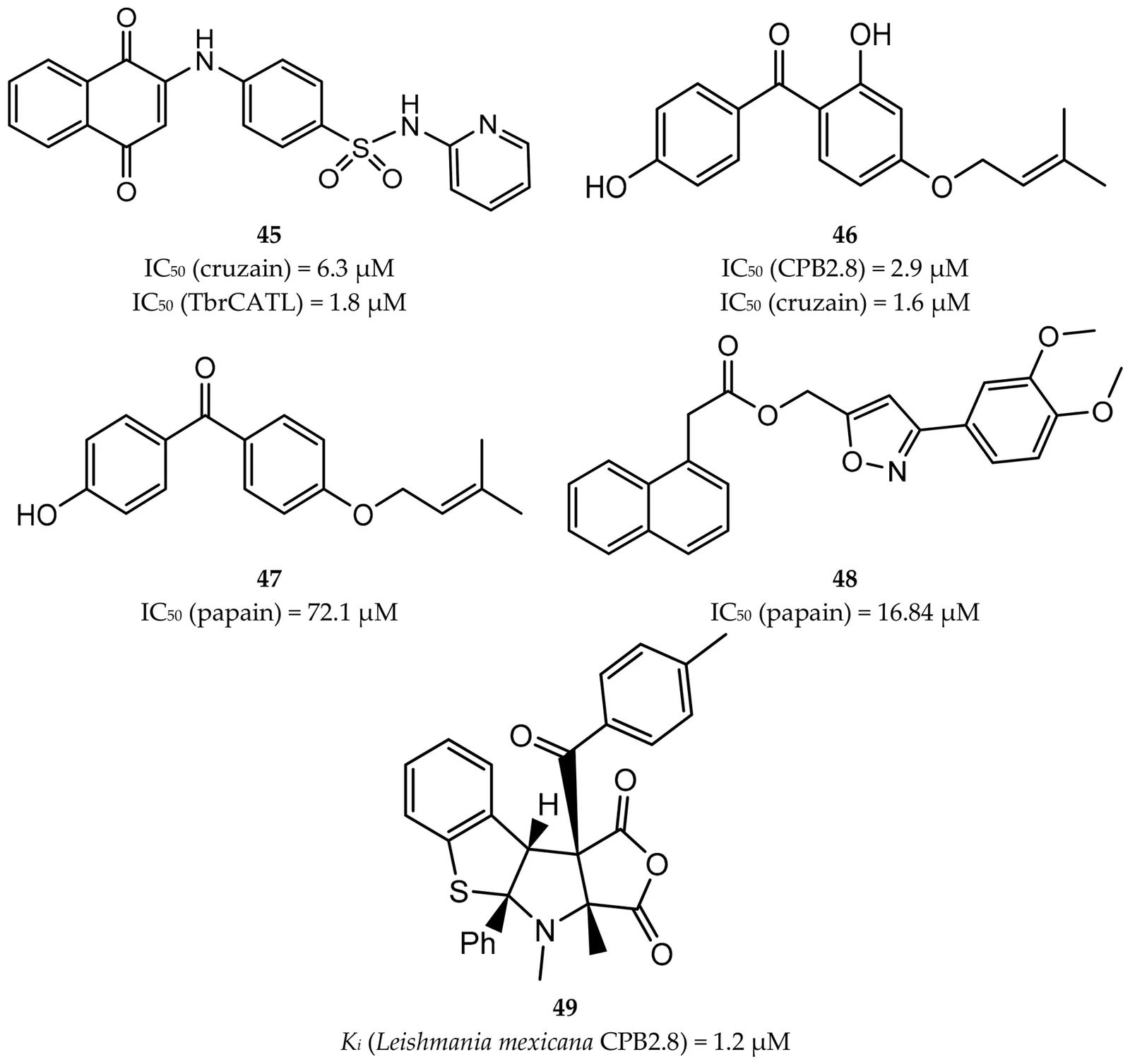
Other oxygen-containing aromatic and naphthalene-based derivatives.

Compounds with a naphthoquinone group are being studied for their CP reversible inhibition [149,166]. For example, compound **45** (Figure 18) is a naphthoquinone-pyridine derivative able to inhibit cruzain (IC_50_ value of 6.3 μM) and rhodesain (IC_50_ = 1.8 μM). At 100 μM, it was able to inhibit 95% of cruzain and 93% rhodesain. However, although both enzymes are crucial for parasite survival, these studies alone are insufficient to draw conclusions about trypanocidal activity. On the one hand, the presence of the quinone group may lead to false-positive results, making it difficult to evaluate its efficacy. On the other hand, the compound needs to reach its target to engage with and inactivate it, which is not demonstrated by the assays performed [166].

Studies have also been made in order to find if benzophenone derivatives are a promising class against *Leishmania* spp. Notably, when tested against the promastigote and the amastigote form of the parasite, they show leishmanicidal activity [179]. In this class of molecules, compound **46** is one of the most promising ones. However, despite presenting lower cytotoxicity than the known drug amphotericin B, used in human treatment of leishmaniasis, it still exhibits an SI of only 6.7. The compound was tested against the recombinant form of *L. mexicana* CPB2.8, inhibiting the enzyme with an IC_50_ value of 2.9 µM. The compound also presented some activity toward cruzain (IC_50_ = 1.6 µM). However, although it was classified as a non-competitive inhibitor, its affinity for both enzymes was relatively low (*K_i_* values > 14 µM). Moreover, its activity against the amastigote form of *Leishmania amazonensis* was only observed at a very high concentration (IC_50_ = 74.4 µM) indicating limited antiparasitic potency [134].

From the same class of compounds, compound **47** was also identified. This compound exhibits a higher SI (11.9). However, it exhibited weak inhibitory activity against papain (IC_50_ = 72.1 µM), and its IC_50_ toward the amastigote form of *L. amazonensis* was 27.4 µM, a much higher value compared to the minimal inhibition value established for *L. donovani* (IC_50_ < 10 µM) [129,134,136]. These findings underscore the importance of developing inhibitors with high selectivity for the target enzyme but also highlight the difficulty in finding them. Moreover, they indicate that the benzophenone warhead might not be a promising scaffold to pursue [134].

Recently, the isoxazole scaffold led to the identification of potent CP inhibitors against *L. donovani*, with compound **48** emerging as the most promising one due to its high potency against the amastigote form (EC_50_ = 1.753 µM), low cytotoxicity toward THP-1 cells (CC_50_ = 35.45 µM), and an SI greater than 20. This compound was able to inhibit papain (IC_50_ = 16.84 µM) and molecular dynamics studies suggest that it might interact with *Leishmania major* CPB, while exhibiting high predicted human intestinal absorption. However, the tested compounds were inhibitors of P-glycoprotein, which might induce drug resistance, a major challenge when developing a drug for widespread use [173].

Lastly, a cyclic anhydride with a lateral ketone group compound (compound **49**), which first acts reversible and later as an irreversible inhibitor, has been found with great selectivity toward *Leishmania mexicana* CPB.2.8, exhibiting an IC_50_ value of 3.7 µM and 90% inhibition at 20 µM. At the same concentration, the inhibition of hCatL was only 30%, revealing its selectivity. Moreover, the compound presents a *K_i_* value of 1.2 µM in the reversible stage, making it a particularly promising candidate against CPB2.8 [180].

All these findings suggest that different scaffolds can be explored as lead structures for the development of CP inhibitors. However, in order to reduce the time and resources required for the research and development process, a careful evaluation must be performed to identify the main advantages of each class of compounds and the structural features that should be optimized. Moreover, a substantial part of this investigation process can be performed using computational approaches, thereby reducing the need to rely exclusively on the traditional synthesis and testing strategy.

### 7.4. Prodrugs

As mentioned before, to overcome bioavailability problems, the development of prodrugs, such as the antiparasitic metronidazole, is a common technique used by the pharmaceutical industry to achieve better correlations between pharmacokinetic and pharmacodynamic properties that can be explored in order to find drugs with trypanocidal and leishmanicidal activity [181,182].

**Figure 19:**
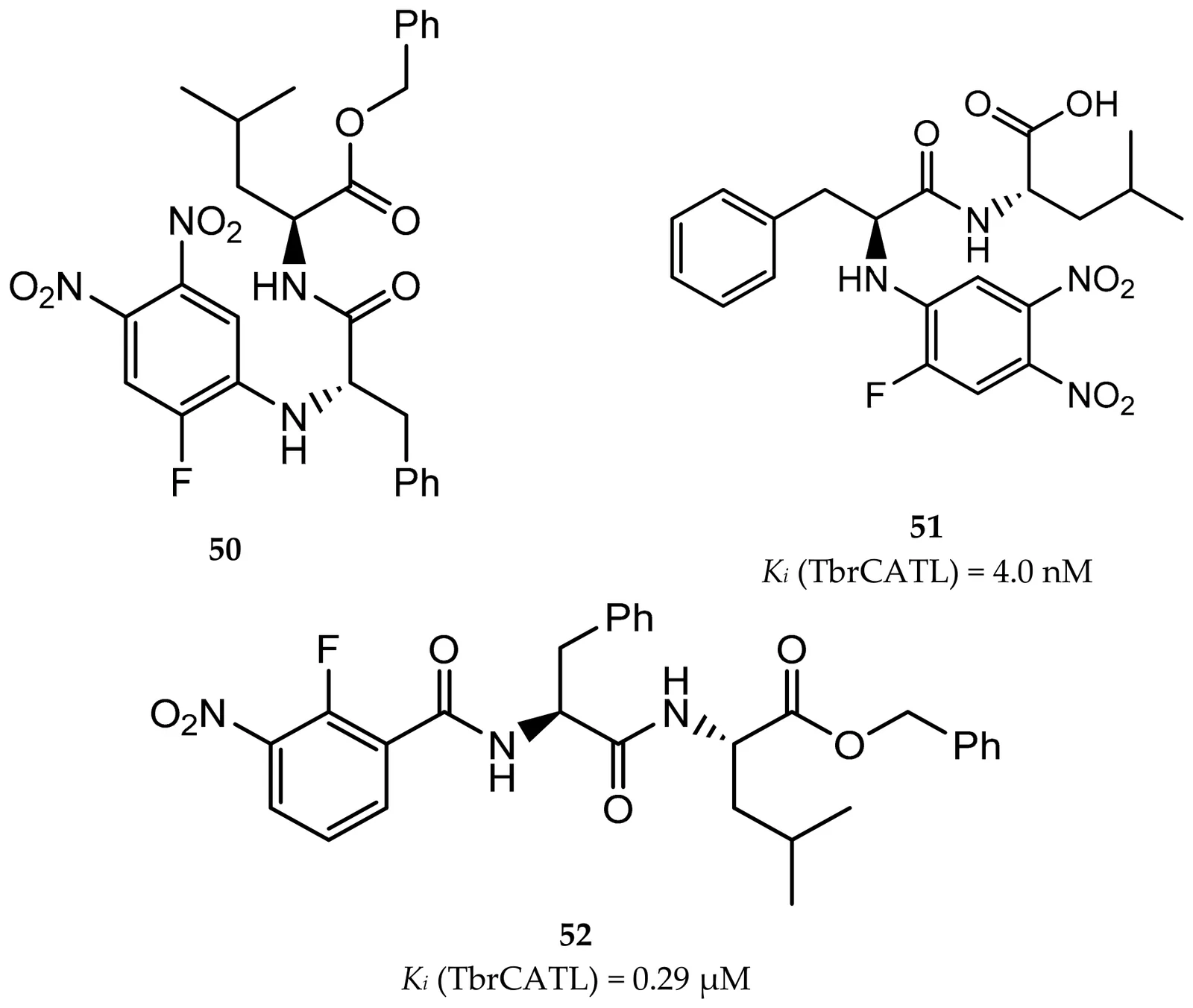
Structures of compounds **50**–**52** and the *K_i_* values of compounds **51** and **52** toward TbrCATL.

Klein and colleagues investigated the activity of peptidic inhibitors bearing the nitro-benzenic group, a class already discussed in this article, and synthesized compounds **50**–**52**, three derivatives with selectivity toward cathepsin proteases and excluded promiscuous inhibition (Figure 19) [183]. However, the noteworthy aspect of this study is the discovery of compound **50** as a prodrug. This compound only inhibited rhodesain in 45% at 20 μM and was more active againts hCatL and hCatB (*K_i_* values of 1.6 μM and 4.4 μM, respectively). Nevertheless after hydrolysis of the ester linkage, compound **50** originates compound **51**, which presents a *K_i_* value of 4.0 nM against rhodesain, a small value when compared to its *K_i_* toward cathepsin L and cathepsin B (3.0 μM and 1.5 μM, respectively). However, the EC_50_ of the benzyl ester compound (**50**) was significantly lower in comparison to the acid compound (**51**), ranging from 95.3 nM to 18.7 μM, probably due to the inability of compound **51** to penetrate the cell membrane. In addition, compound **52**, capable of inhibiting 75% of TbrCATL activity at 20 μM (*K_i_* = 0.29 μM), was also identified. However, the affinity of the compound toward the target was equipotent to the hCatL (*K_i_* = 0.66 μM), making the prodrug approach the most compelling therapeutic strategy identified by the investigators [183].

### 7.5. Archived Library Boxes Compounds

#### 7.5.1. Compounds Found Using the Malaria and Pathogen Databox

**Figure 20:**
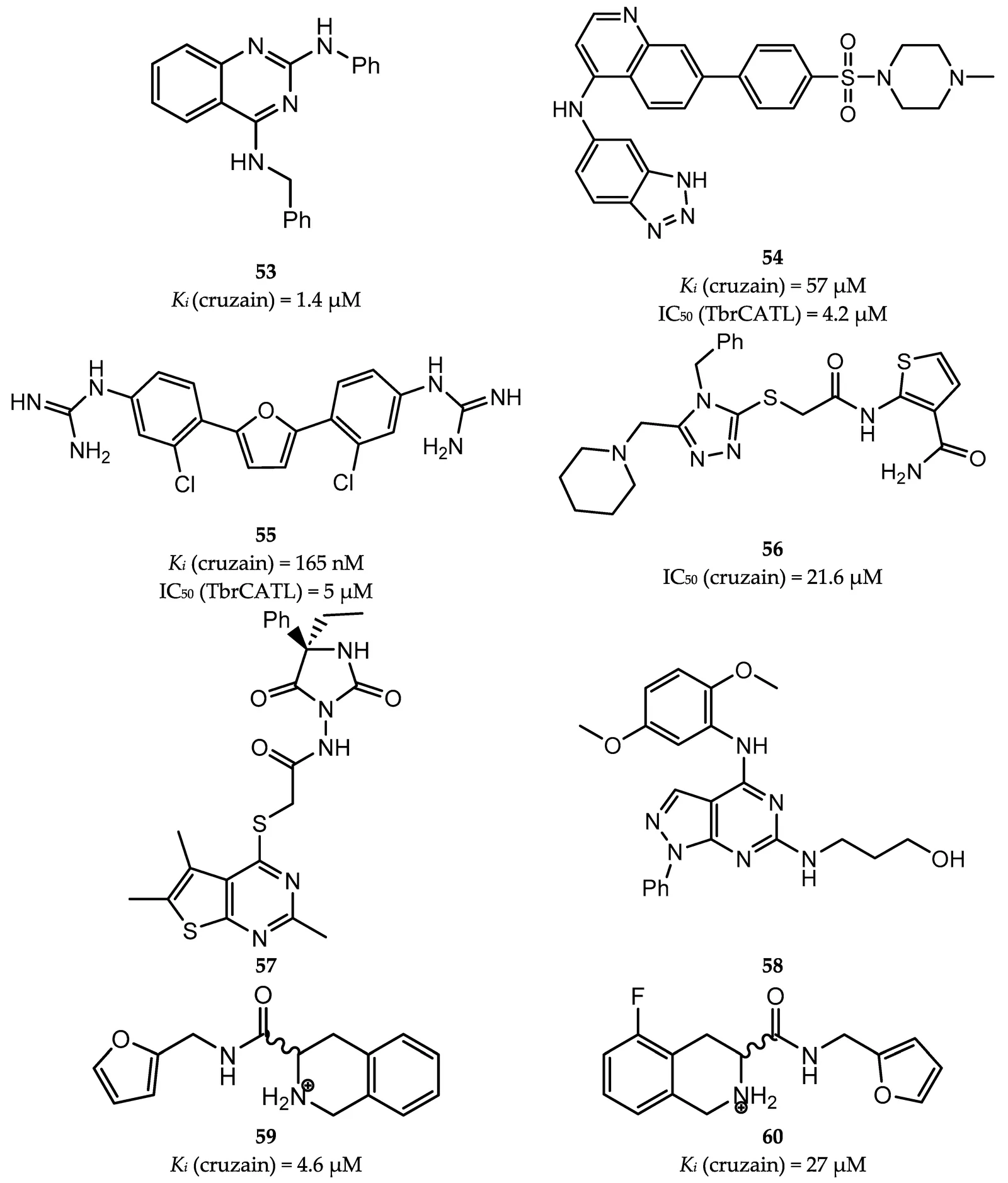
Compounds obtained from libraries that act as CP inhibitors.

Using the Malaria Box, specifically the MMV400 set, Pereira and Da Silva identified compound **53** (Figure 20) which is able to inhibit cruzain and rhodesain in a competitive way [184]. Enzymatic assays further confirmed its potency, yielding an IC_50_ value of 9 μM and *K_i_* value of 1.4 μM against cruzain, in addition to lower activity toward rhodesain (IC_50_ = 30 µM). Although compound **53** exhibited some sensitivity in the detergent assay, this effect was observed only at concentrations considerably higher than that required to inhibit cruzain (50 µM). Moreover, this compound demonstrated antiparasitic activity, proved by its IC_50_ value of 1.5 μM against *T. cruzi*, 3.4 μM against *T. brucei brucei* and 3.1 μM against *T. brucei rhodesiense*, demonstrating that a single compound can be a possible strategy to combat more than one species responsible for NTDs [184].

Compounds **54** and **55** (Figure 20) are cruzain inhibitors (*K_i_* = 57 µM and *K_i_* = 165 nM, respectively) retrieved from the pathogen databox. Notably, the double chlorine derivative (compound **55**) is able to reduce almost 100% of cruzain activity at 5 µM. Moreover, the compound is also potent against TbrCATL, exhibiting an IC_50_ value of 4 µM. For cruzain, this value is even lower (IC_50_ = 0.46 µM). However, despite the high potency and non-covalent mechanism of action, compound **55** only reached the pre-clinical phase, probably due to its CC_50_ value being around 11.6 µM, which reveals its lack of selectivity toward the parasites [185]. These findings emphasize the challenges in finding candidates that simultaneously exhibit high potency, selectivity and safety for the treatment of diseases caused by kinetoplastid parasites.

#### 7.5.2. Compounds Found Using the ZINC Database

The ZINC database is frequently used as a first step in the identification of new CP inhibitors [186,187,188].

Wiggers and colleagues resorted to this database and to structure–activity relationship (SAR) studies to find compound **56** (Figure 20), a weak cruzain inhibitor (IC_50_ = 21.6 μM) with more potent activity against the trypomastigote of *T. cruzi* (IC_50_ = 10.6 μM), suggesting the existence of more than one mechanism of action. Moreover, its SI was lower than 5, meaning that it would have a very weak safety profile and, even if this value is similar to the current treatment with benznidazole, safer options should be considered [187].

Compounds **57** and **58** were also retrieved from the ZINC database. These compounds are particularly interesting due to their safety profile, since both have a CC_50_ > 200 μM when tested in NCTC cells and a high SI (greater than 14.7 and 19.8, respectively) compared with benznidazole (>10.7). Compound **57** is predicted to bind to the active site of cruzain and exhibited an IC_50_ value of 13.6 μM against *T. cruzi* trypomastigotes, using this enzyme as a target [188]. Nevertheless, the percentage of enzymatic inhibition or the *K_i_* value should be determined to further assess the potential of these molecules as cruzain inhibitors and sustain the interaction of these compounds with the active site of the enzyme, predicted by a molecular-docking strategy [189,190,191].

On the other hand, compound **59** is an enantiomeric molecule that was able to completely inhibit cruzain with an IC_50_ of 3 μM and *K_i_* value of 4.6 μM [192]. The predictions indicate that enantiomer S is more stable than enantiomer R due to its linkage to the active site of cruzain, but racemic mixtures were used for the enzymatic test. Regarding the in vivo activity of compound **59**, this molecule was mostly active against *T. cruzi* epimastigotes, which reduces its relevance in Chagas disease’s therapeutic options since this form is only present in the vector. In contrast, the same team also discovered compound **60**, which was active against the trypomastigote and amastigote forms, the ones present in the host’s organism. Indeed, this derivative was able to reduce the parasite release and parasite load, reducing the intracellular replication without damaging the macrophages. Moreover, it inhibited cruzain with an IC_50_ of 26 μM and with a *K_i_* value of 27 μM. Consequently, considering that neither compounds **59** or **60** were flagged for PAINS moieties or were active againts hCatL at 100 μM, they could represent a promising hit for further optimization, notwithstanding the lack of toxicity and pharmacokinetic studies. Furthermore, this also highlights the importance of characterizing the compounds under study in order to determine whether the differences in activity are due to different mechanisms of action or whether the activity of each enantiomer critically influences trypanocidal activity [192].

### 7.6. Derivatives of Natural Compounds

Other strategies that scientists use to discover new drugs use natural plant- or animal-based compounds [193,194,195].

#### 7.6.1. Gallinamide A and Its Derivative

**Figure 21:**
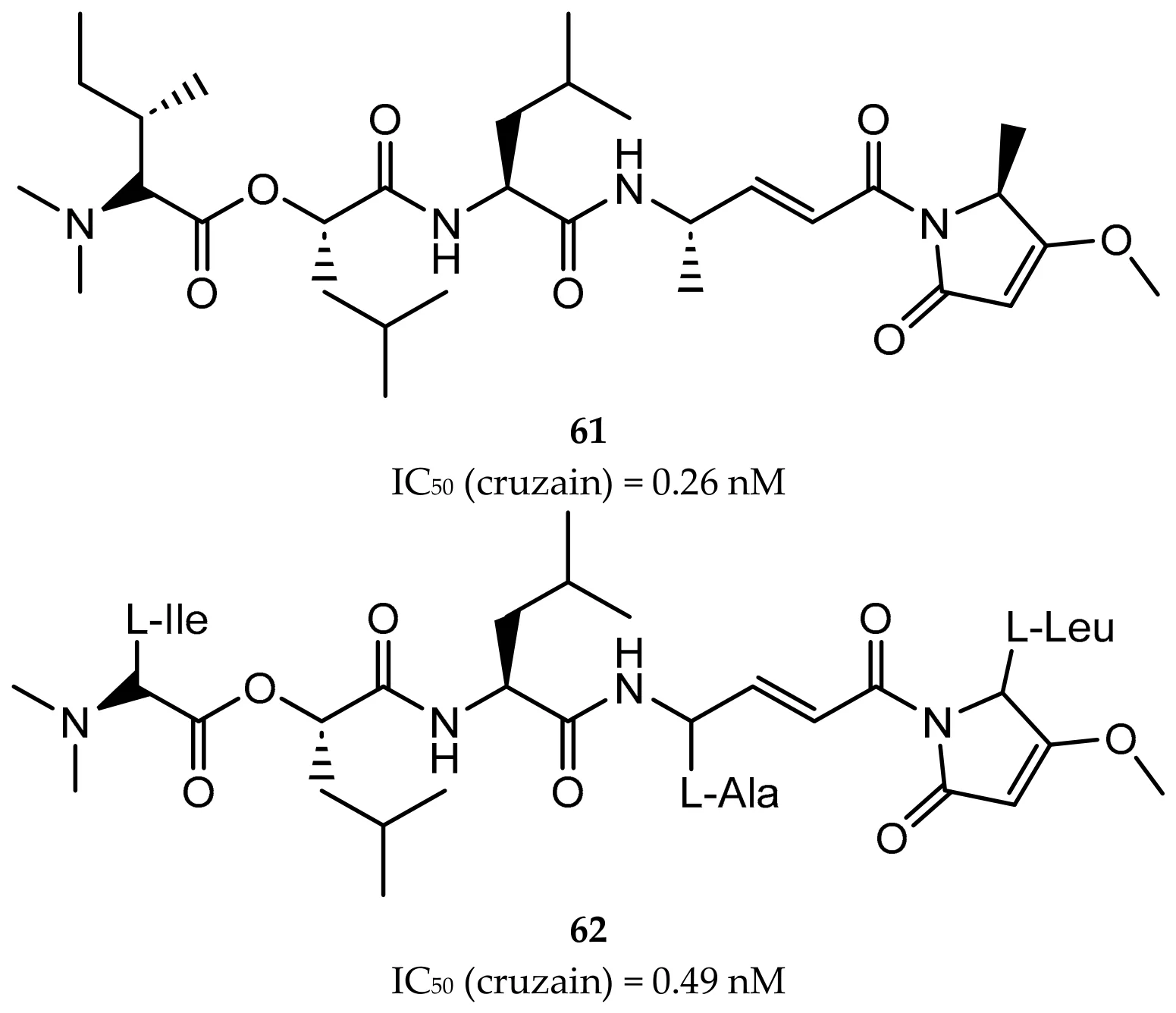
Structures of compounds **61** and **62** and their respective IC_50_ values against cruzain.

Gallinamide A, identified as compound **61** (Figure 21), was first discovered as an antimalarial agent and later identified as a potent inhibitor of hCatL and other cathepsin L-like CPs [135,196,197]. This compound was revealed to be potent against cruzain (IC_50_ = 0.26 nM), while capable of having a great trypanocidal activity, being active against the amastigote form of *T. cruzi* (LD_50_ = 14.7 nM). Compound **62** was developed based on the scaffold of this drug, exhibiting an IC_50_ value of 0.49 nM toward cruzain and LD_50_ value of 5.1 nM against the parasite. Moreover, neither compound **61** nor **62** showed cytotoxicity toward murine cells, even at concentrations of 10 μM [135]. These results are important because even though both compounds inhibited the hCatL with high affinity (*K_i_* = 4.67 nM and *K_i_* = 1.73 nM for compounds **61** and **62**, respectively), they were still non-toxic to host cells, confirming that inhibition of hCatL is not inherently fatal, even though it can compromise hormonal function [135,198].

#### 7.6.2. Chalcone and Flavonoid Derivatives and Curcumin

**Figure 22:**
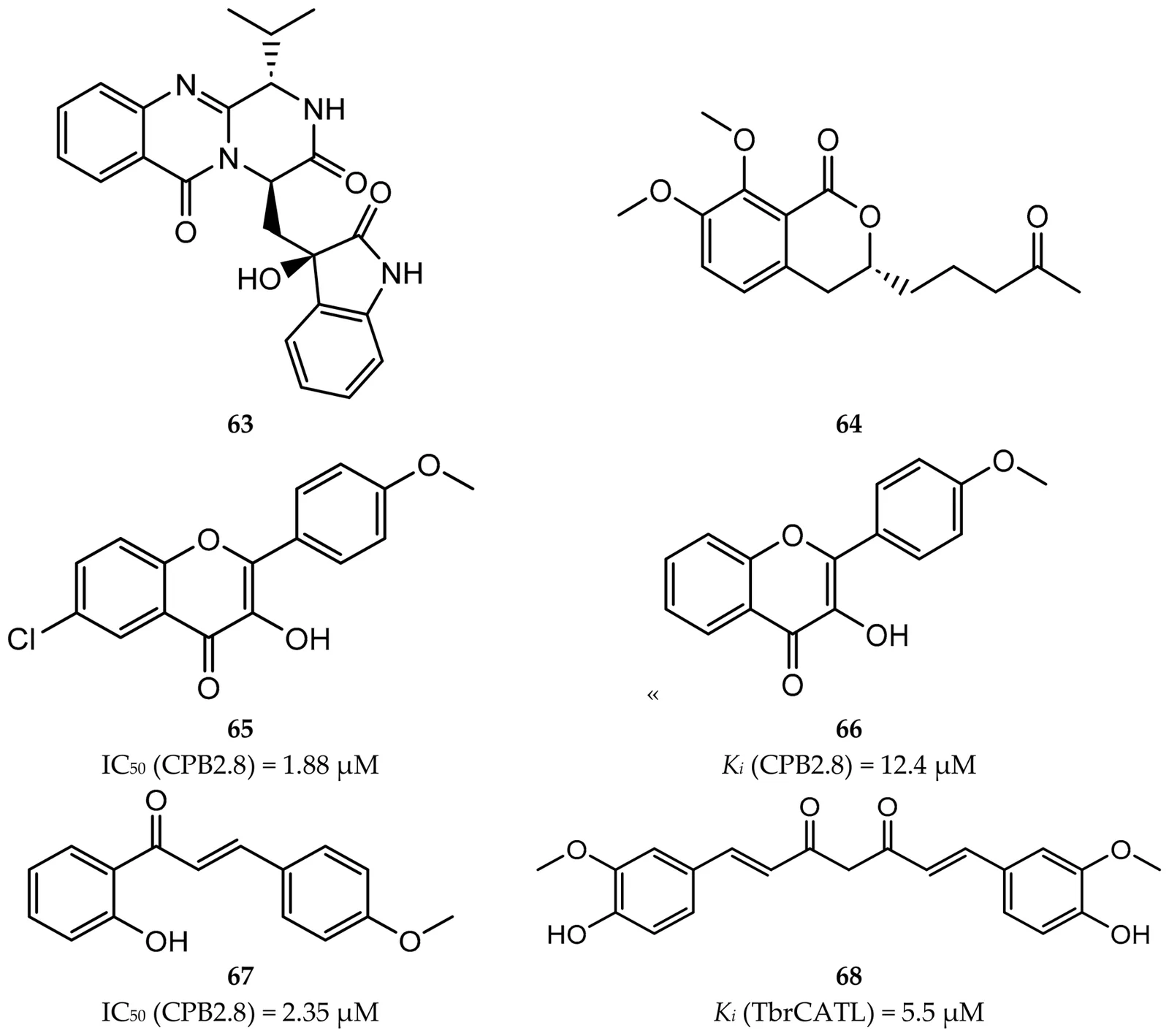
Derivatives obtained from natural compounds.

Extracts from plants and fungi are also commonly used as a first step in drug discovery [195,199,200]. The ethanolic extract of rosemary root-derived *Talaromyces* sp. and *Cladosporium* sp. led to the identification of compounds **63** and **64** (Figure 22), respectively, with potential inhibition of rhodesain. The most potent extract against *T. brucei* was the one obtained from *Cladosporium* sp. (IC_50_ = 1.3 µg/mL) [195]. However, given the magnitude of these diseases, the identification of a promising extract is merely the preliminary stage in drug development. Its pursuit is imperative to isolate the specific bioactive compounds and to characterize both their enzymatic inhibition profile and trypanocidal efficacy.

For *Leishmania* spp., flavonoid derivatives have been under investigation as CPB inhibitors, with some of them being particularly potent. Compound **65** exhibited an IC_50_ value of 1.88 µM against the recombinant form of CPB2.8 from *L. mexicana* and an IC_50_ value of 1.08 µM toward the extracellular form of *L. amazonensis*, reinforcing the hypothesis that CPB inhibition may lead to leishmanicidal activity. Moreover, the SI is greater than 764.70, revealing it to be a potent compound with a great security margin. In the same class of compounds, compound **66** was also identified with a *K_i_* value of 12.4 µM toward CPB2.8 but no activity against the parasite, suggesting that the chlorine group might be important for the leishmanicidal activity of flavonoid derivatives. Among the chalcone derivatives identified, the most potent compound tested against the enzyme (IC_50_ = 2.35 µM) was compound **67**, which was also active against the promastigote form of the parasite (IC_50_ = 0.71 µM) and presented an SI of 1324. These observations suggest that these scaffolds represent a promising starting point for the discovery of selective compounds active against leishmaniasis. However, further biological evaluation against the amastigote form and additional cytotoxic tests need to be made [201].

Another example of natural products serving as primary scaffolds in drug discovery is curcumin (Figure 22—compound **68**), originally obtained from *Curcuma longa*, since its derivatives remain under active investigation, in particular in the antiparasitic field [154,194,202,203]. Steverding and colleagues identified compound **68** as a reversible rhodesain inhibitor with a *K_i_* value of approximately 5.5 μM and IC_50_ value of 5.6 μM against the enzyme [203]. However, discrepancies in the reported potency of curcumin persist, with reported IC_50_ toward rhodesain ranging frequently from around 7 μM to more than 20 μM [141,154,202]. These heterogenous results might be attributable to variations in the purity of the compound and diverse experimental parameters. Furthermore, the intrinsic chemical instability of curcumin complicates the reproducibility of the results. Additionally, the clinical translation of curcumin is severely restricted due to its pharmacokinetic properties. In particular, its poor aqueous solubility hinders its action as a potential drug, although a new set of formulations is being studied to improve its bioavailability and facilitate administration [194,204].

### 7.7. Combined Therapies

**Figure 23:**
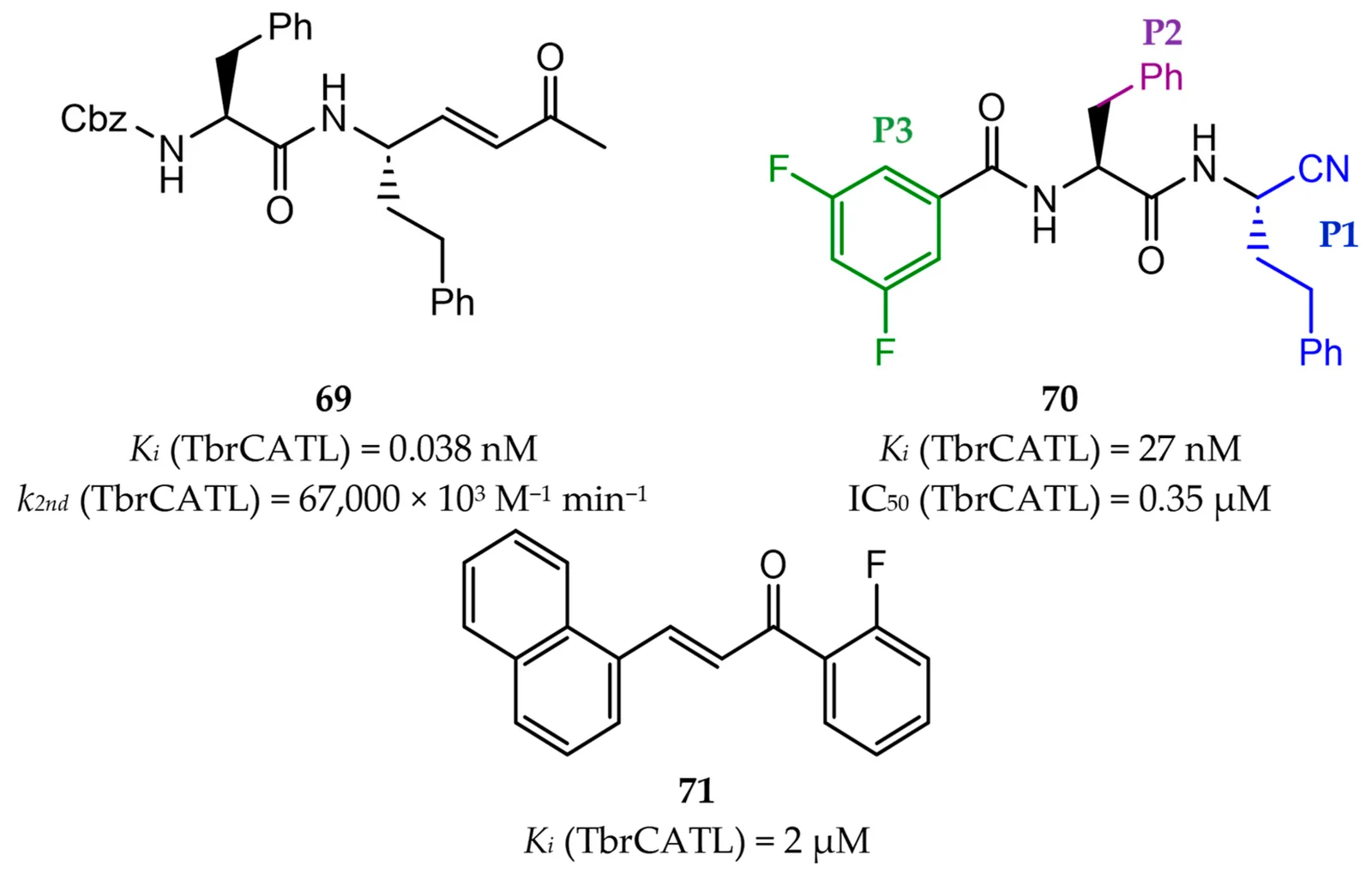
Structure and main enzymatic activity of compounds **69**, **70** and **71**.

Di Chio and colleagues have contributed significantly to the field of combination therapies against trypanosomatids [141,154,202]. In this line of work, they studied a series of curcumin derivatives and found compound **69** (Figure 23), a very potent (IC_50_ = 9 nM, *K_i_* = 0.038 nM and *k_2nd_* = 67,000 × 10^3^ M^−1^ min^−1^) and irreversible inhibitor of rhodesain with IC_50_ value of 2.33 μM toward *T. brucei brucei* [202,205]. However, they were pursuing a combination strategy using compound **69** and curcumin. This approach did not reduce IC_50_ values of either compound, indicating that successful combination therapy requires more than simply combining two potent compounds acting with potential synergistic activity, even though combination therapy may reduce potential toxic effects [154,202]. Di Chio and co-workers also synthesized compound **70**, with enhanced selectivity toward TbrCATL, yielding a *K_i_* value of 27 nM and SI of 7.26 when compared to the hCatL (*K_i_* = 196 nM). Although this dipeptidyl nitrile is structurally similar to compound **16**, it is slightly less potent, given its IC_50_ value (0.35 μM) against the TbrCATL compared to the IC_50_ value from the first derivative (compound **16**—IC_50_ = 0.2 μM) toward the same enzyme. Nevertheless, its improved selectivity profile marks a significant advancement in minimizing potential off-target effects. Compound **70** was also tested in concomitant administration with curcumin, and, in this case, the drug combination resulted in 100% inhibition of rhodesain and increased selectivity toward the enzyme, indicating potent synergism between the compounds and a more effective combination [141].

Following the same strategy, compound **71**, a chalcone derivative with a *K_i_* of 2 µM against rhodesain and *K_i_* of 20 mM toward hCatL, was also identified and combined with curcumin, resulting in a synergistic effect that enhanced enzyme inhibition. This strategy is important because it may reduce the minimum effective dose, diminishing the probability of cytotoxicity and, therefore, being a possible approach to achieve a better selectivity and safer profile for drugs [206].

## 8. Conclusions and Future Perspectives

Among the most studied classes of compounds, natural product-derived molecules (including derivatives of curcumin, Gallinamide A, and flavonoids), as well as compounds containing Michael acceptors, nitrile and thiosemicarbazone moieties seem to be the most promising, considering their potency and capability of inhibiting the different enzymes while maintaining activity toward the parasites.

The vinyl sulfone group, known for its ability to irreversibly inhibit CPSs through a Michael addition mechanism, can confer nanomolar potency to peptidic inhibitors toward these enzymes. Compounds **3** and **4** exemplify this effect, exhibiting very low *K_i_* values against TbrCATL. However, although these compounds could represent significant advances in the development of antiparasitic agents, their clinical potential would depend on an adequate safety profile, for which no information is currently available [80]. Nevertheless, based on the example of K11777, some lack of selectivity and, consequently, potential toxicity may be expected from these compounds.

Peptidic inhibitors bearing the nitro-benzenic group have also been the focus of recent studies. Compounds **10** and **11** are the most potent derivatives of this class, exhibiting nanomolar *K_i_* values against TbrCATL [144]. However, the potential use of compound **50** as a prodrug, exploiting its lipophilic properties to facilitate the delivery of the active metabolite (compound **51**), represents a very important strategy worth exploring, particularly toward *Leishmania* spp., in which the drug most achieves the amastigote form and therefore crosses both host and parasite cell membranes [183].

Furthermore, among peptidic inhibitors, special relevance should be given to dipeptidyl nitrile derivatives, since these compounds are able to inhibit the main CPs of *T. cruzi*, *T. brucei* and *Leishmania* spp. with nanomolar potency, making them one of the most promising classes of compounds for the development of a single inhibitor capable of targeting multiple parasites [121,152,153].

The thiosemicarbazone scaffold has also been under scrutiny over the last few years for its CP inhibition properties. Indeed, this class of derivatives includes compounds active against cruzain and TbrCATL, with demonstrated trypanocidal activity toward amastigotes and trypomastigotes, representing another possibility for the strategy of combating multiple NTDs with a single drug [77,131,146,171].

However, the potency of the already discovered compounds is still above the expected values for a compound to be considered a hit. Therefore, further optimization of their structure is still needed. However, according to the established criteria for evaluating the therapeutic potential of hit compounds in the treatment of infectious diseases, Gallinamide A and its derivatives, compounds **61** and **62**, respectively, were identified as the most promising candidates for the treatment of Chagas diseases, owing to their great inhibition of cruzain (IC_50_ < 1 µM), combined with strong trypanocidal activity (IC_50_ < 10 µM) and no cytotoxicity exhibited in mammals’ cells [129,135,136].

For HAT, urea derivative containing a Michael acceptor group (compound **2**), the tripeptide (compound **23**) and a derivative from curcumin (compound **69**) are the most potent TbrCATL inhibitors. However, despite their strong potency against TbrCATL, all three compounds exhibit IC_50_ values above the threshold generally considered indicative of promising anti-*T. brucei brucei* activity, highlighting the discrepancy between enzyme inhibition and trypanocidal efficacy [120,129,136,159,202].

For the treatment of leishmaniasis, a flavonoid derivative (compound **65**) is considered the most promising candidate. This compound inhibits *L. mexicana* CPB2.8 with an IC_50_ well below 10 µM, the threshold generally considered acceptable for the treatment of *L. donovani* infection, and exhibits an outstanding SI (>100) [129,136,201]. An aziridine compound (compound **43**) is also promising against the intracellular amastigote stage of the parasite, exhibiting an IC_50_ substantially lower than the value typically expected for a hit compound and an SI above 29. Furthermore, this compound inhibits both CPB2.8 and CPC which may explain its remarkable activity [48,129,136].

The search for selective inhibitors of cruzain, rhodesain and CPB, the major CPs of *T. cruzi*, *T. brucei* and *Leishmania* spp., has evolved considerably over the years. The research has progressed from irreversible to reversible inhibitors, including peptidic derivatives, scaffold-based drug design, natural product-derivative compounds, and crossing different strategies to increase selectivity and improve pharmacokinetic properties. This continuous shift in medicinal chemistry approaches has led to the abandonment of several compound classes that, despite extensive investigation, failed to provide conclusive evidence of antitrypanosomal activity or a clear path toward clinical development. On the one hand, exploring diverse chemical scaffolds and design strategies broadens the opportunities to identify novel and effective inhibitors. On the other hand, the lack of sustained optimization within individual chemical series and the dispersion of research efforts across numerous classes of compounds may contribute to the delay in finding effective therapies for these NTDs.

To summarize, Chagas disease, HAT and leishmaniasis remain critical diseases for which there are limited effective therapeutic options, underscoring the urgent need for the development of novel trypanocidal agents and drug delivery systems capable of efficiently delivering these compounds to their site of action.

Moreover, considerable progress has been achieved in the field of CP inhibitors over the past decades, with different strategies to identify selective compounds being explored, including database screening, the use of prodrugs, and combined therapeutic strategies. However, several challenges remain in their development for the treatment of trypanosomatid diseases. Overcoming these challenges will require a multidisciplinary approach that integrates medicinal chemistry, biochemistry, pharmacology, and parasitology to optimize inhibitor potency, selectivity, pharmacokinetic properties, and in vivo efficacy.

Lastly, the future of protein targeting may lie not only in protein inhibition but also in targeted protein degradation, with selective inhibitors serving as potential scaffolds for the development of PROTAC-like molecules. However, to the best of our knowledge, no significant advances have yet been made toward the development of a true PROTAC as antiparasitic agents, as many aspects of the ubiquitin-mediated protein degradation system of the parasites remain poorly understood or are still under investigation. Therefore, unlike in other areas of microbiology, the development of a true protein degrader targeting cruzain, TbrCATL or CPs from *Leishmania* spp. may still require substantial research efforts. Nevertheless, continued efforts to optimize these inhibitor scaffolds are warranted, both to improve their intrinsic antiparasitic activity and to explore their potential contribution to the design of future protein degraders.

## Figures and Tables

**Table 1:** In vitro potency and cytotoxicity profile of the lead dipeptidyl compounds **14** and **15**.

Compound	p*K_i_* Cruzain	p*K_i_* CPB	p*K_i_* hCatB	p*K_i_* hCatL	EC_50_ *T. cruzi* (µM)	CC_50_ LLCMK_2_ Cells (µM)	Ref.
**14**	7.5	7.4	5.1	8.3	4.1	97.9	[153]
**15**	7.2	6.8	6.3	8.1	4.9	>100	[153]

Abbreviations: CPB, cysteine protease B from *Leishmania mexicana*; hCatB, human cathepsin B; hCatL, human cathepsin L.

**Table 2:** Enzymatic activity of peptidyl enoates.

Compound	IC_50_ Cruzain	IC_50_ TbrCATL	IC_50_ hCatB	IC_50_ hCatL	*k_2nd_* Cruzain	*k_2nd_* TbrCATL	*k_2nd_* hCatB	*k_2nd_* hCatL	Ref.
**18**	3.75	1.13	1.13	2.45	3600	36,700	4200	1900	[155]
**19**	0.08	0.145	0.05	0.26	60,800	116,200	42,400	29,900	[155]

IC_50_ in μM units; *k_2nd_* in M^−1^s^−1^ units. Abbreviations: CPB, cysteine protease B from *Leishmania mexicana*; hCatB, human cathepsin B; hCatL, human cathepsin L.

**Table 3:** Enzymatic and biological activity of peptidic compounds bearing the vinyl ketone group.

Compound	*K_i_* TbrCATL (nM)	*k_2nd_* TbrCATL (M^−1^ min^−1^)	EC_50_ *T. brucei brucei* (µM)	Ref.
**23**	0.66	21,000,000	2.3	[159]
**24**	8.04	17,000,000	-	[159]

**Table 4:** Enzymatic and in vitro activities of compounds **25** and **26**.

Compound	*K_i_* Cruzain (µM)	*K_i_* hCatB (µM)	*K_i_* hCatL (µM)	EC_50_ *T. cruzi* (µM)	EC_50_ *T. brucei brucei* (µM)	Ref.
**25**	0.171	6.5	1.9	5.9	4.0	[76]
**26**	0.126	37	0.88	5.4	2.8	[76]

Abbreviations: hCatB, human cathepsin B, hCatL, human cathepsin L.

**Table 5:** Thiosemicarbazones as inhibitors of cruzain and TbrCATL.

Compound	IC_50_ TbrCATL (µM)	IC_50_ Cruzain (µM)	EC_50_ *T. cruzi* (µM)/% Inhibition	Ref.
**33**	0.230	0.176	-	[146]
**34**	-	0.00286	-	[146]
**35**	2.0	14.4	5.5 µM (amastigotes)	[77]
**36**	0.0218	0.2001	52% (amastigotes, 50 μM)	[131]
**37**	0.698	0.1446	57% (amastigotes, 50 μM)	[131]
**38**	-	0.306	1.96 µM (trypomastigotes)	[171]

## Data Availability

No new data were created or analyzed in this study. Data sharing is not applicable to this article.

## Abbreviations

The following abbreviations are used in this manuscript:

BBB	Blood–Brain Barrier
CL	Cutaneous Leishmaniasis
CP	Cysteine Protease
HAT	Human African Trypanosomiasis
hCatB	Human Cathepsin B
hCatL	Human Cathepsin L
ML	Mucocutaneous Leishmaniasis
NTD	Neglected Tropical Disease
PAINS	Pan-Assay Interference Compounds
POI	Protein of Interest
PROTAC	Proteolysis Targeting Chimera
SAR	Structure–Activity Relationship
SI	Selectivity Index
VL	Visceral Leishmaniasis
VSG	Variant Surface Glycoprotein
WHO	World Health Organization
